# Melastatin family Transient Receptor Potential channels support spermatogenesis in planarian flatworms

**DOI:** 10.1387/ijdb.240180lr

**Published:** 2025

**Authors:** HALEY NICOLE CURRY, ROGER HUYNH, LABIB ROUHANA

**Affiliations:** 1 Department of Biological Sciences, Wright State University, Dayton, OH, USA,; 2 Department of Biology, University of Massachusetts Boston, Boston, MA, USA

**Keywords:** spermatogenesis, planarian, Transient Receptor Potential Channel Melastatin, TRPM

## Abstract

The Transient Receptor Potential superfamily of proteins (TRPs) form cation channels that are abundant in animal sensory systems. Amongst TRPs, the Melastatin-related family (TRPMs) is composed of members that respond to temperature, pH, sex hormones, and various other stimuli. Some TRPMs exhibit enriched expression in the gonads of vertebrate and invertebrate species, but their contributions to germline development remain to be determined. We identified twenty-one potential TRPMs in the planarian flatworm *Schmidtea mediterranea* and analyzed their anatomical distribution of expression by whole-mount *in situ* hybridization. Enriched expression of two TRPMs (*Smed-TRPM-c* and *Smed-TRPM-l*) was detected in testis, whereas eight TRPM genes had detectable expression in patterns representative of neuronal and/or sensory cell types. Functional analysis of TRPM homologs by RNA-interference (RNAi) revealed that disruption of normal levels of *Smed-TRPM-c* expression impaired sperm development, indicating a role for this receptor in supporting spermatogenesis. *Smed-TRPM-l* RNAi alone did not result in a detectable phenotype, but it did increase sperm development deficiencies when combined with *Smed-TRPM-c* RNAi. Fluorescence *in situ* hybridization revealed expression of *Smed-TRPM-c* in early spermatogenic cells within testes, suggesting cell-autonomous regulatory functions in germ cells for this gene. In addition, *Smed-TRPM-c* RNAi resulted in reduced numbers of presumptive germline stem cell clusters in asexual planarians, suggesting that *Smed-TRPM-c* supports the establishment, maintenance, and/or expansion of spermatogonial germline stem cells. While further research is needed to identify the factors that trigger Smed-TRPM-c activity, these findings reveal one of the few known examples for TRPM function in the direct regulation of sperm development.

## Introduction

Studies of animal reproduction are relevant for preservation of natural resources, raising livestock, forestry, fishery, and protection of crops from pests. Environmental influences, such as temperature, are known to alter endogenous endocrine pathways that regulate seasonal reproduction either directly by serving as a cue for activation developmental processes, or indirectly by altering metabolic pathways that influence fecundity (Chmura and Williams, 2022). As climate change continues to threaten the survival of hundreds of species, our need to understand how organisms sense changes in environmental conditions, as well as the molecular mechanisms behind their influence on mating, fertility, and fecundity is of outmost urgency.

Planarian flatworms are free-living members of the superphylum Lophotrochozoa that are best known for their regenerative abilities. Although fission and regeneration serve as means for asexual reproduction of some planarians, most species reproduce sexually as cross-fertilizing hermaphrodites (Hyman, 1951, Vila-Farré and Rink, 2018; Issigonis and Newmark, 2019). Planarians that live in habitats with fluctuating temperatures have been reported to switch reproductive modes seasonally (Kenk, 1935; Nodono and Matsumoto, 2022). Sexualization can also be induced experimentally in the laboratory (Kobayashi *et al*., 2004), and the signaling molecules that ignite or modulate sexual maturation are starting to be identified (Collins *et al*., 2010; Kobayashi *et al*., 2017; Rouhana *et al*., 2017; Nakagawa *et al*., 2018; Issigonis *et al*., 2023). Furthermore, reproductive structures regress in response to injury or starvation, and redevelop upon healing and growth, showing extreme developmental plasticity (Newmark *et al*., 2008).

Given their dynamic reproductive biology, planarians provide a great opportunity to learn about the molecular mechanisms behind external influence on sexual reproduction. The sexual organs and entire reproductive system of planarians, including the germline, develop post-embryonically from somatic pluripotent stem cells called neoblasts (Kobayashi *et al*., 1999; Wang *et al*., 2007). Planarians lack gonads at birth, but these develop during growth in individuals whose genetic composition and environmental conditions allow for sexual maturation. In the planarian *Schmidtea mediterranea*, germline stem cells are observed quickly after birth in dorsolateral testis primordia that extend from the tail to the area immediately posterior to the head (Wang *et al*., 2007). Germline stem cells in testis primordia are observed before ovarian counterparts appear, which corroborates with development of male gonads prior to development of ovaries during growth as sexual maturation occurs in this species (reviewed by Issigonis and Newmark, 2019).

Planarians continuously replenish, grow, and regenerate their entire anatomy through differentiation of neoblasts (Newmark and Sanchez Alvarado, 2002). Given that expression of genes can be disrupted systemically by supplementing food with double-stranded RNA (dsRNA) of target-specific sequence (Rouhana *et al*., 2013), interruption of continuous cellular turnover allows the discovery of genes required for development of tissues (including the germline). Systemic RNAi in planarians also allows for identification of genes required during sexual maturation and development of reproductive structures without interfering with embryonic development. This approach has uncovered novel genes required for germline development, as well as previously unknown roles in gametogenesis for genes characterized in other systems (Wang *et al*., 2010; Rouhana *et al*., 2017; Saberi *et al*., 2016; Khan and Newmark, 2022).

This study includes characterization of genes predicted to encode Transient Receptor Potential (TRP) channels of the Melastatin family (TRPM) in the planarian *S. mediterranea*. TRPMs mediate cellular responses to environmental and physiological cues that include hormones, changes in pH, and temperature (Diver *et al*., 2022; Zheng, 2013). Mammalian TRPMs that respond to changes in temperature are expressed in somatosensory neurons, where they play roles in noxious and innocuous temperature response (Kashio and Tominaga, 2022). However, some members of the TRPM family are also expressed during mammalian spermatogenesis, where they are thought to influence sperm development and function (Martínez-López *et al*., 2011; De Blas *et al*., 2009; Lee *et al*., 2003; Borowiec *et al*., 2016). Here we demonstrate that one of the TRPM homologs in *S. mediterranea*, *Smed-TRPM-c*, displays testis-specific expression and is required to maintain normal capacities of spermatogenesis, providing an avenue to study the contributions of TRPMs to regulation of sperm development in planarians. In asexual planarians, *Smed-TRPM-c* is required for normal maintenance of presumptive germline stem cells, suggesting a function in the earlier stages of spermatogenesis. Altogether, these results complement findings in mice to indicate that TRPMs may have an ancestral role during spermatogenesis and suggest that TRPMs may regulate spermatogenesis in response to changes in temperature.

## Results

### Identification of Transient Receptor Potential Melastatin (TRPM) family channels in *Schmidtea mediterranea*

To identify putative TRPMs in the planarian flatworm *S. mediterranea*, TBLASTN searches for homologs of human TRPM3 (NCBI GenBank ID NP_066003.3) were performed against transcriptomes of sexual *S. mediterranea* deposited in PlanMine (the planarian flatworm sequence repository; Brandl *et al*., 2016; Rozanski *et al*., 2019). Hits were further analyzed by reciprocal BLAST searches against annotated human proteins. Twenty-one non-redundant putative TRPM genes were identified (Supplementary Table S1). Analysis of identical sequences by BLASTN in reference transcriptomes of the asexual biotype of *S. mediterranea* revealed matches for all 21 putative TRPM genes (Supplementary Table S1), indicating that these are expressed at levels detectable by RNAseq in asexual planarians. Three of these genes are orthologs of TRPMs previously characterized in the planarian *Dugesia japonica* (Inoue *et al*., 2014). The genes represented by contigs dd_Smed_v6_17857_0_1 and dd_Smed_v6_26481_0_1 on PlanMine are orthologs of DjTRPMa and were therefore named *Smed-TRPM-a1* and *Smed-TRPM-a2*, respectively (BLASTP E-values = 0.0 and 2.04 × 10^−135^). The gene represented by contig dd_Smed_v6_9288_0_1 was named *Smed-TRPM-b*, as it is orthologous to DjTRPMb (E-value = 0.0). The remaining 18 TRPM homologs in *S. mediterranea* were named *Smed-TRPM-c* to -*t*. The sequence of *Smed-TRPM-a* to -*e*, and four additional genes, more closely matched TRPM3 than any other human TRPM in reciprocal BLAST results, while others more closely matched human TRPM1, 2, 4, 5, 6, and 8 (Supplementary Table S1). These results indicate an expansion of TRPM genes to at least 21 homologs in *S. mediterranea*.

### *Smed-TRPM-c* and *Smed-TRPM-l* are expressed in testis lobes of *S. mediterranea*

**Fig. 1.**
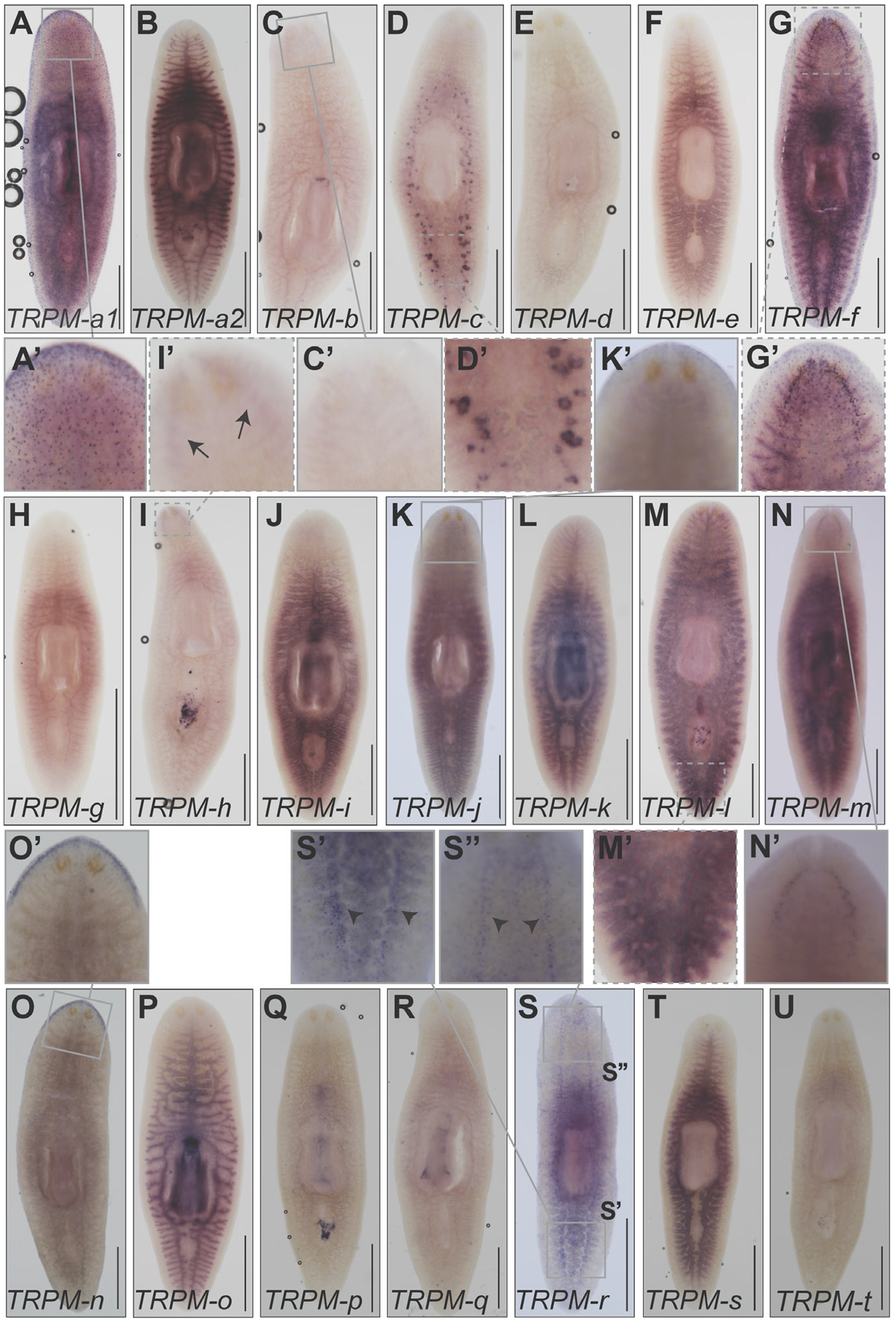
Analysis of expression of TRPMs in *S. mediterranea* reveals two homologs expressed in testes. **(A-U)** Whole-mount colorimetric *in situ* hybridization analyses using partial sequence riboprobes (~500 nts) display distribution of expression of *Smed-TRPM-a1* (*TRPM-a1*; A), *Smed-TRPM-a2* (*TRPM-a2*; B), and *Smed-TRPM-b* to -*t* (*TRPM-b* to -*t*; C-U). Insets show magnified view of planarian testis lobes in the posterior dorsal region of planarians (D’ and M’), as well as sensory cells (A’ and G’), brain region (C’, G’, I’, N’), head periphery (K’ and O’), and cells along the posterior (S’) and anterior (S”) ventral nerve cords. Arrows point at the location of brain lobes in (I’), and arrowheads point at posterior ventral nerve cords in (S’ and S”). Scale bars, 1 mm.

To determine the anatomical distribution of expression of planarian TRPM genes in *S. mediterranea*, whole-mount *in situ* hybridization (WMISH) analyses were performed using sexually mature planarian hermaphrodites. Using riboprobes that corresponded to ~500 bps of reference cDNA contig sequence (Supplementary File S1), *Smed-TRPM-a1* expression was detected in cells concentrated at the head tip and further distributed throughout the planarian body (Fig. 1A), matching the expression of its ortholog *DjTRPMa* (Inoue *et al*., 2014) in thermosensory neurons. *Smed-TRPM-a2* expression was only detected in the gut (Fig. 1B). Expression of *Smed-TRPM-b*, -*f*, -*h*, and -*m* was detected in the brain region (Fig. 1 C,G,I,N), with WMISH signal for some of these genes being limited to subsets of putative neurons (Fig. 1 G’,N’) or very faint (Fig. 1 C’,I’). *Smed-TRPM-c* expression was only detected in testis lobes, which are abundantly located from the tail to the region posterior to the head along the dorsolateral anatomy of *S. mediterranea* (Fig. 1 D,D’). Expression of *Smed-TRPM-j* and -*n* were observed along the periphery of the head tip (Fig. 1 K,O), whereas *Smed-TRPM-r* expression was enriched in subsets of cells that reside along the ventral nerve cords and brain (Fig. 1 S,S”). *Smed-TRPM-e*, -*f*, -*o*, and -*s* expression was detected through much of the gut (Fig. 1 F,G,P,T), while expression of *Smed-TRPM-l* was detected in the gut (Fig. 1M) and testis (Fig. 1M’). Expression of *Smed-TRPM-c* and *Smed-TRPM-l* in testes was validated through single and double fluorescence *in situ* hybridization (FISH), which revealed partial overlap in expression of these genes in the outer layer of testis lobes (Supplementary Fig. S1). *Smed-TRPM-o* was detected in the gut and pharynx (Fig. 1P). It is important noting that the planarian gut and copulatory apparatus tend to generate background signal in WMISH analyses using colorimetric development, therefore some of the signals from these structures were deemed inconclusive (Supplementary Table S1). The TRPM homologs with no detected or inconclusive expression include *Smed-TRPM-d*, -*g, -i*, -*k*, -*p*, -*q*, and -*t* (Fig. 1 E,H,J,L,Q,R,U).

### *Smed-TRPM-c* is required to maintain normal levels of sperm development

**Fig. 2.**
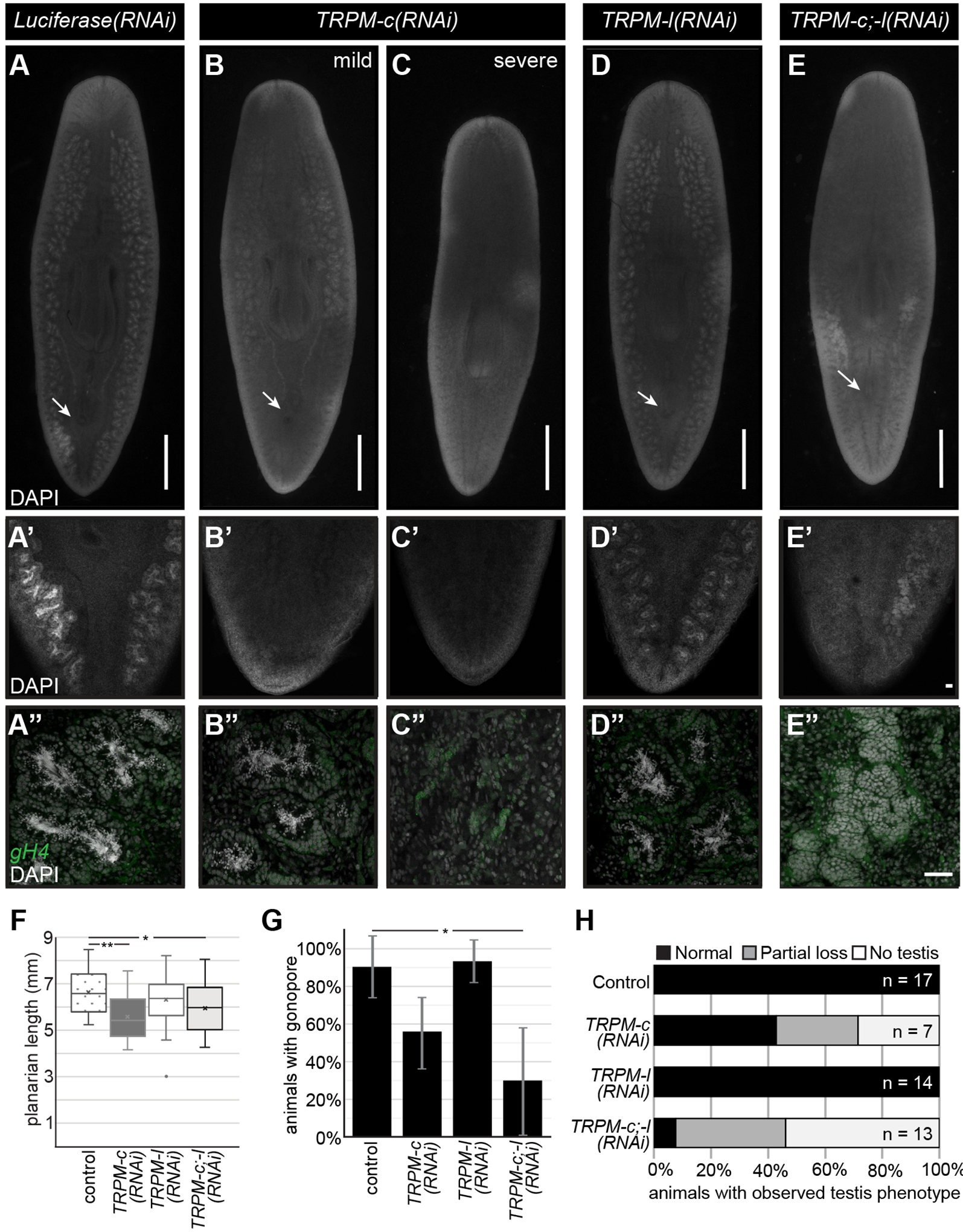
Decreased testis development is observed upon *Smed-TRPM-c* knockdown and *Smed-TRPM-c;TRPM-l* double knockdown. **(A-E)** DAPI signals from whole-mount staining of *Luciferase(RNAi)* (A-A”) and *Smed-TRPM-l(RNAi)* (D-D”) show normal distribution of testis lobes along the dorsolateral anatomy of sexual planarians (A,D). Accumulation of spermatozoa inside testis lobes can be observed in these same samples by confocal microscopy under 10X (A’, D’) and 20X (A”, D”) objectives. Parallel analysis of *Smed-TRPM-c(RNAi)* and *Smed-TRPM-c;TRPM-l(RNAi)* (*TRPM-c;-l(RNAi)*) show partial (B; E) to complete (C) loss of testis lobes and/or testis lobes producing spermatozoa (B”-C”, and E”). Germline stem cells and spermatogonia in testes are shown using *germinal histone H4* (*gH4)* fluorescence *in situ* hybridization signal (green in A”-E”). Development of testis lobes observed in *Luciferase(RNAi)* and *Smed-TRPM-l(RNAi)* posterior regions (A’ and D’) was particularly reduced in *Smed-TRPM-c(RNAi)* and *Smed-TRPM-c;TRPM-l(RNAi)* samples (B’-C’, and E’). **(F)** Box and whisker plot showing average planarian length post-fixation. Mean is marked by an “X”. **(G)** Percentage of animals in each RNAi group displaying a fully developed copulatory apparatus at end of RNAi treatment. Graphs indicate averaged values from three different experiments with error bars representing standard deviation. **(H)** Quantification of testis development phenotypes (as in A-E) in animals of size comparable to sexually mature controls illustrate percentage of samples with normal testis development (black bar), as well as partial (gray bar) and complete (white bar) absence of testis in each group. Arrows indicate position of copulatory organ in (A-E). Scale bars, 1 mm (A-E) and 50 μm (E’ and E”). Statistical significance in (F) and (G) is indicated by one asterisk (*) in comparisons for which Student’s *t*-test *p* < 0.05, and by two asterisks (**) where *p* < 0.01.

To test whether TRPMs expressed in planarian testes are required for sperm development, juvenile (sexually immature) planarian hermaphrodites were subjected to continuous RNAi treatments in groups targeting either *Smed- TRPM-c* (*Smed-TRPM-c(RNAi)*), *Smed-TRPM-l* (*Smed-TRPM-l(RNAi)*), or both *Smed-TRPM-c* and *Smed-TRPM-l* (*Smed-TRPM-c;TRPM-l(RNAi)*). Planarians were fed dsRNA corresponding to ~500 bps of gene-specific sequence (Supplementary File S1) mixed in their food twice per week. A control group was fed dsRNA with sequence that corresponds to a fragment of firefly *Luciferase* sequence, which does not interfere with planarian homeostasis or germline development (Magley and Rouhana, 2019; Lesko and Rouhana, 2020; Christman *et al*., 2021). After six weeks of continuous RNAi treatment, planarians grew and were fixed and stained with 4’,6-diamidino-2-phenylindole (DAPI) to visualize the anatomy and distribution of testis lobes. As expected, testis lobes were abundant along the dorsolateral anatomy of *Luciferase(RNAi)* (Fig. 2A), and the same was seen for *Smed-TRPM-l(RNAi)* planarians (Fig. 2D). On the other hand, testis lobes were partially or completely missing in regions of *Smed-TRPM-c(RNAi)* and *Smed-TRPM-c;TRPM-l(RNAi)* planarians (Fig. 2 B–C,E). The absence of testis lobes was particularly noticeable at the posterior end of these animals (Fig. 2 B’–C’,E’), which is a region where testis lobes are normally abundant (Fig. 2 A’,D’). Beyond distribution of testis lobes, analysis of sperm development by high magnification confocal microscopy revealed full progression of spermatogenesis and spermatozoa accumulation in the innermost region of testis lobes of *Luciferase(RNAi)* and *Smed-TRPM-l(RNAi)* planarians (Fig. 2 A”,D”). In contrast, *Smed-TRPM-c(RNAi)* and *Smed-TRPM-c;TRPM-l(RNAi)* planarians had testis lobes with disruptions at different stages of spermatogenesis. These included loss of spermatozoa (Fig. 2B”), loss of spermatids and/or spermatocytes (Fig. 2E”), and reduced spermatogonia (Fig. 2C”).

Sexual maturation and development of reproductive structures are related to growth and somatic integrity (Kobayashi *et al*., 1999; Wang *et al*., 2007). Therefore, animal size and development of somatic reproductive structures were analyzed in *Smed-TRPM-c*, *Smed-TRPM-l*, and *Smed-TRPM-c;TRPM-l* knockdowns. *Smed-TRPM-c(RNAi)* displayed reduced average animal length when compared to the *Luciferase(RNAi)* control and *Smed-TRPM-c(RNAi)* groups (Fig. 2F). Additionally, about half of the *Smed-TRPM-c(RNAi)* as well as *Smed-TRPM-c;TRPM-l(RNAi)* planarians failed to develop copulatory structures (Fig. 2G), indicating compromised sexual maturation. To assess whether the reduced testis phenotype in *Smed-TRPM-c(RNAi)* resulted from size deficiencies, sperm development was compared between animals of equivalent size or larger than the smallest sexually mature planarian from the *Luciferase(RNAi)* group (*i.e*., exhibiting fully developed copulatory structures). Amongst these groups, *Smed-TRPM-c(RNAi)* planarians still displayed spermatogenesis defects not observed in the control or *Smed-TRPM-l(RNAi)* groups, and knockdown of *Smed-TRPM-l* in the *Smed-TRPM-c;TRPM-l(RNAi)* group seemed to exacerbate the loss of testis and sperm development (Fig. 2H). These results suggest that *Smed-TRPM-c* is required for testis development and/or spermatogenesis directly, and that the spermatogenesis phenotype of Smed-TRPM-c(RNAi) is not a consequence of growth defects. Interestingly, levels of *Smed-TRPM-l* RNA were more robustly decreased by RNAi than *Smed-TRPM-c* RNA levels (75% and 70% average decrease for *Smed-TRPM-l* 1-week into RNAi treatments for single and double target gene knockdown, respectively, vs. 20% and 15% for *Smed-TRPM-c*; Supplementary Fig. S2), which may explain the variability of phenotypic penetrance observed upon *Smed-TRPM-c* RNAi.

### *Smed-TRPM-c* is preferentially expressed in developing sperm and required to maintain sperm production

**Fig. 3.**
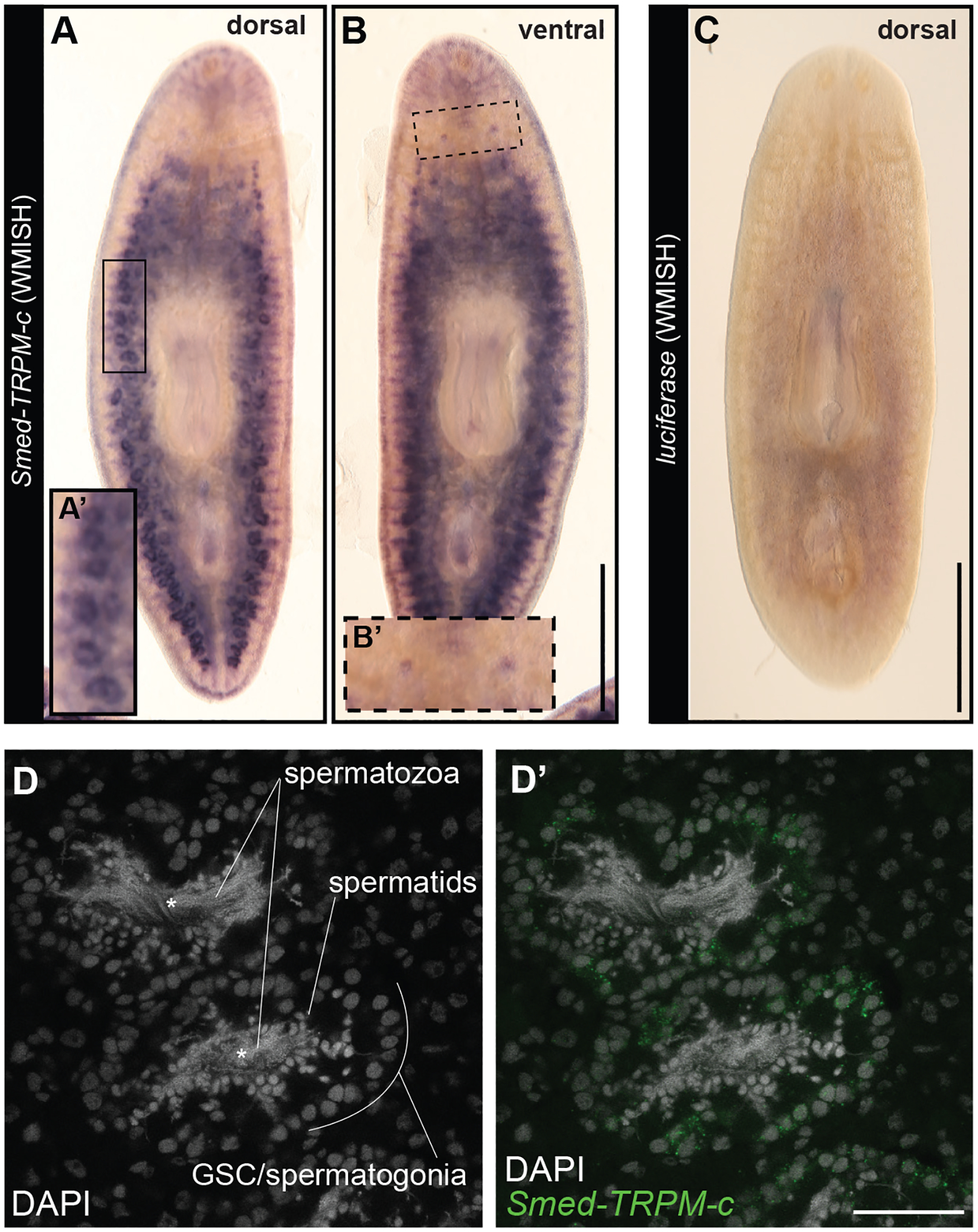
*Smed-TRPM-c* is preferentially expressed in testes. **(A-C)** Whole-mount *in situ* hybridization analysis using full-length ORF probes in sexual planarians shows enriched expression of *Smed-TRPM-c* in testis lobes of sexual planarians (n = 8/8; A) and in the ventrally located ovaries (n = 2/8; B). Background signal generated from negative control *Luciferase* riboprobes is shown in (C). Insets show 2-fold magnified views of testis lobes (A’; full frame inset) and ovaries (B’; dashed frame). **(D)** Confocal section displaying the signal detection of *Smed-TRPM-c* fluorescence *in situ* hybridization, green **(D’)** in the outer cellular region of testis lobes. DAPI stain of nuclear DNA (gray; D and D’) show changes in cellular morphology during the progressive differentiation of spermatogenic cells from the outer to inner region of each lobe. Asterisks mark location of the innermost region of each testis lobe. Scale bar, 1 mm in A-C, and 50 μm in D’.

Given that disruption of *Smed-TRPM-c* expression alone resulted in phenotypic observations comparable to *Smed-TRPM-c;TRPM-l* double knockdown, further studies were carried out focusing on *Smed-TRPM-c*. The full cDNA sequence for *Smed-TRPM-c* was obtained by 3’ and 5’ rapid amplification of cDNA ends (RACE), followed by direct amplification, cloning, and sequencing of *Smed-TRPM-c* open reading frames. Expression of *Smed-TRPM-c* was analyzed using riboprobes generated from full-length ORF clones, which confirmed preferential expression in testis lobes (Fig. 3 A,A’; n = 8/8 planarians) and validated WMISH results observed using partial sequence (Fig. 1D). Interestingly, expression of *Smed-TRPM-c* was also detected in the ovaries in a fraction of our samples when using full-length riboprobes (Fig. 3 B,B’; n = 2/8 planarians). Non-specific signal from WMISH generated from parallel analyses using a riboprobes against firefly *Luciferase* as negative control was minimal (Fig. 3C), confirming the specificity of *Smed-TRPM-c* signals. Expression of *Smed-TRPM-c* was not conclusively detected outside of the gonads, although faint signals were observed in regions of the gut and head (Fig. 3 A,B). However, single cell RNAseq (scRNA-seq) data available for asexual planarians (Plass *et al*., 2018; Fincher *et al*., 2018), failed to support the notion that *Smed-TRPM-c* is expressed in the gut or in neurons (Supplementary Fig. S3). In fact, there is no corroboration regarding detection of expression of *Smed-TRPM-c* in any somatic cell type between different scRNA-seq studies (Plass *et al*., 2018; Fincher *et al*., 2018; Zeng *et al*., 2018), although one indicated comparably higher expression in glia than is other somatic tissues (Plass *et al*., 2018; Supplementary Fig. S3). These results indicate that *Smed-TRPM-c* is preferentially expressed in the testes.

Fluorescent *in situ* hybridization was performed using full-length ORF riboprobes to further assess the cellular distribution of *Smed-TRPM-c* expression in testes. Planarian testis lobes are primarily populated by germ cells undergoing spermatogenesis (Wang *et al*., 2007; Newmark *et al*., 2008; Collins *et al*., 2010). Spermatogonial stem cells proliferate to form cysts that differentiate progressively as their location changes from periphery to center of each testis lobe (*i.e*., germline stem cells/spermatogonia in the outer region, and spermatids and spermatozoa in the innermost region of the lobe; Fig. 3D). *Smed-TRPM-c* expression was detected most abundantly in the periphery of the testis lobe, which is where male germline stem cells and spermatogonia are located (Fig. 3D’). These results indicate that *Smed-TRPM-c* is preferentially expressed during early stages of spermatogenesis.

**Fig. 4.**
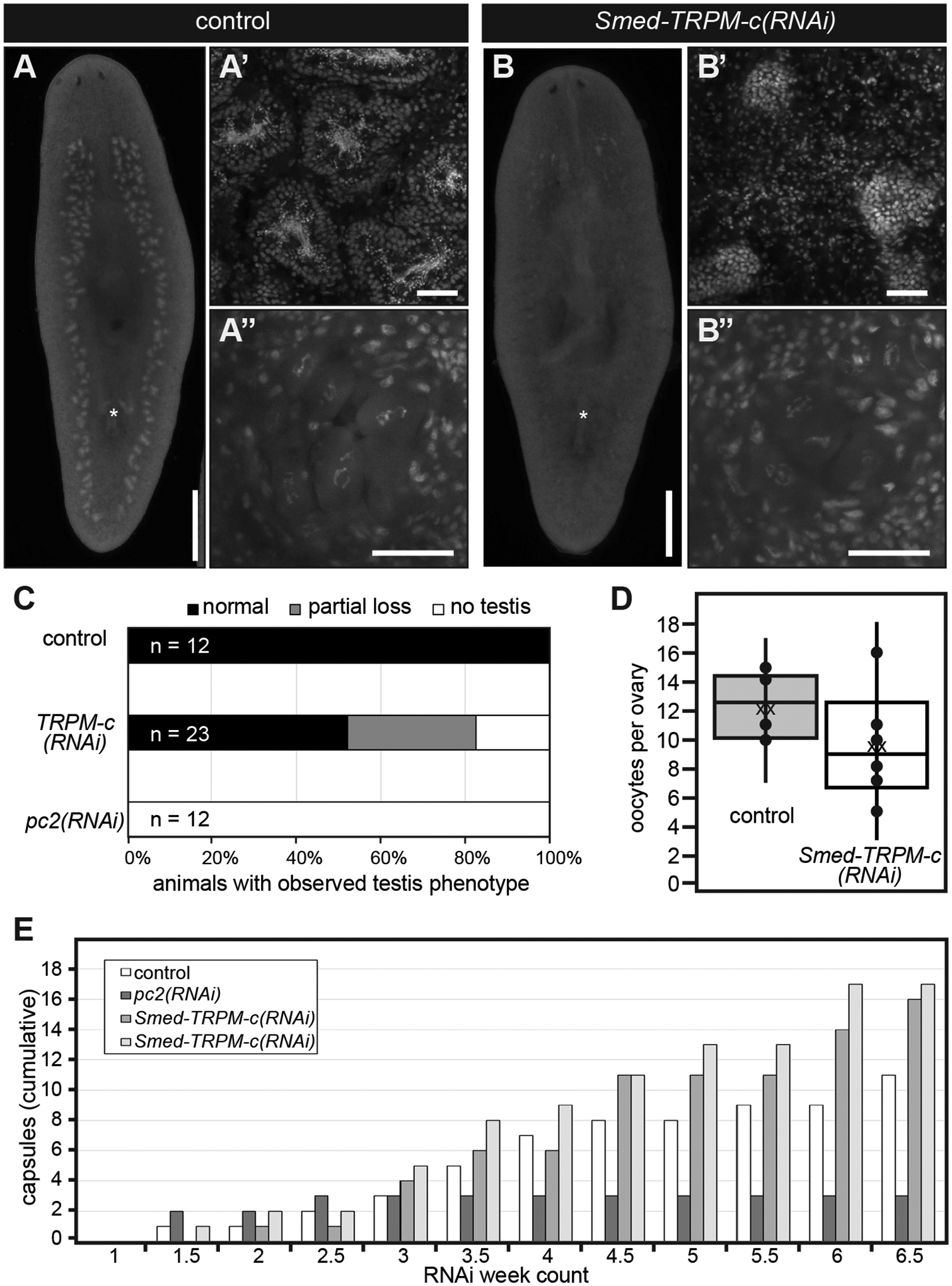
*Smed-TRPM-c* supports sperm development. **(A,B)** Analysis of DAPI-stained samples by whole-mount fluorescence stereomicroscopy reveals testis lobe distribution and presence of copulatory apparatus (asterisks) in *Luciferase(RNAi)* (control; A) and *Smed-TRPM-c(RNAi)* (B) planarians. Analyses of same samples by single-plane confocal microscopy reveal normal sperm development in *Luciferase(RNAi)* planarians **(A’)** and sperm development disruption in *Smed-TRPM-c(RNAi)* **(B’)**, whereas development of oocytes seems comparable **(A” and B”)**. Scale bars, 1 mm (A,B) and 50 μm (A′-B′ and A”-B”). **(C,D)** Quantification of gonad development phenotypes (as in A,B) revealed normal testes distribution (black bar; C) in all control samples, and partial (gray bar) or complete loss (white bar) of productive testes in almost half of all *Smed-TRPM-c(RNAi)* planarians (n = 23). Complete loss of testes was observed in all *pc2(RNAi)* planarians (n = 12). **(D)** Box and whisker plot show a slight decrease in the number of oocytes per ovary in *Smed-TRPM-c(RNAi)* samples (white) when compared to *Luciferase(RNAi)* (gray). This difference was not statistically significant (*t*-test, *p* > 0.05). Dots represent individual data points, “xx” marks the mean. **(E)** Analysis of cumulative egg capsule production as a readout of functional somatic reproductive structures reveals continuous production of capsules in *Luciferase(RNAi)* (control, white bars) and two groups of *Smed-TRPM-c(RNAi)* planarians (lighter shades of gray), whereas *pc2(RNAi)* planarians (dark gray) ceased producing capsules 2.5 weeks into RNAi treatments.

RNAi experiments performed using dsRNA corresponding to full-length *Smed-TRPM-c* ORF were performed to verify the phenotype observed upon knockdown of this gene using partial sequence. Groups of juvenile *S. mediterranea* were fed dsRNA mixed with liver twice per week, and a positive control group for RNAi efficacy (*Smed-prohormone convertase 2; pc2(RNAi)*) along with a negative control group (*Luciferase(RNAi)*) were included for comparison. After six weeks of RNAi, samples of comparable size were stained with DAPI and analyzed by fluorescence stereo and confocal microscopy. DAPI staining revealed normal testis morphology and distribution in all of the *Luciferase(RNAi)* samples (n = 12; Fig. 4 A–A’,C). As was expected from previous studies (Collins *et al*., 2010), animals from the *pc2(RNAi)* group lacked testes and showed progressively less motility (Fig. 4C; not shown), indicating that the RNAi regimen was effective. Interestingly, about half of the animals in the *Smed-TRPM-c(RNAi)* group (n = 23) displayed fewer and underdeveloped testes in comparison to control samples (Fig. 4 B–B’,C). In contrast, analysis of ovaries did not reveal noticeable changes in morphology between *Luciferase(RNAi)* and *Smed-TRPM-c(RNAi)* (Fig. 4 A”,B”), and quantification of the number of oocytes present per ovary did not reveal statistically significant a difference in *Smed-TRPM-c(RNAi)* and control planarians (Fig. 4D; mean 12.2 oocytes/ovary in *Luciferase(RNAi)* vs. 9.6 oocytes/ovary *Smed-TRPM-c(RNAi)*; unpaired two-tailed student’s *t*-test, p > 0.05). These results indicate that *Smed-TRPM-c* is primarily required to support normal sperm development in planarians.

To further check for pleiotropic defects related to maintenance of sexual maturation, production of egg capsules was analyzed in sexually mature *Smed-TRPM-c(RNAi)* planarians. Planarians, like most flatworms, produce ectolecithal eggs that lack the nutritional components necessary for early development (Gremigni, 1983). Instead, the embryo is encapsulated with yolk cells produced by yolk glands (vitellaria) that develop at the end of sexual maturation using modifications to oogenic genetic programs (Rouhana *et al*., 2017; Issigonis *et al*., 2022). Importantly, production and deposition of egg capsules occur independently of presence or absence of functional gametes (Steiner *et al*., 2016). To test the requirement of *Smed-TRPM-c* in vitellaria maintenance and function, sexually mature planarians were subjected to six weeks of RNAi treatments and the number of egg capsules deposited was recorded after each dsRNA feeding as an indicator of vitellaria presence and function. The number of capsules deposited by *Luciferase(RNAi)* control planarians accumulated continuously over the course of RNAi treatment (Fig. 4E) as previously observed (Rouhana *et al*., 2017; Steiner *et al*., 2016). In contrast, *pc2(RNAi)* planarians produced capsules only during the first two weeks of treatment (Fig. 4E), which was expected given that *pc2* function is required for maintenance of sexual maturation (Collins *et al*., 2010). *Smed-TRPM-c(RNAi)* planarians produced egg capsules continuously over the course of the experiment reaching even higher quantities than the *Luciferase(RNAi)* group (Fig. 4E), indicating that *Smed-TRPM-c* is not required for yolk cell development, maintenance, or function. Hatchling production was not quantified due to difficulties that would emerge from the considerable subset of *Smed-TRPM-c(RNAi)* animals that do produce sperm (Figs. 2H and 4C), as well as the already low and variable hatching rate observed for capsules of laboratory cultures of *S. mediterranea* (22% to 48%; Steiner *et al*., 2016). Nevertheless, the fact that *Smed-TRPM-c(RNAi)* planarians produced capsules as often as control animals indicates that in addition to yolk glands, structures that participate in capsule formation, such as the gonopore and genital atrium, were functional in *Smed-TRPM-c(RNAi)* planarians.

In addition to testing for capsule formation and deposition, changes in behavior were investigated in *Smed-TRPM-c(RNAi)* to assess the possibility of neuronal functions or non-specific knockdown of TRP paralogs with functions in *S. mediterranea* sensory behaviors (Arenas *et al*., 2017; Ross *et al*., 2018; Ross *et al*., 2024). Chemotaxis response was indirectly tested by tracking the fraction of planarians that ate during RNAi feedings, for which no differences were observed (data not shown). To test for changes in behavioral response to temperature, an arena composed of two cold (17°C) and two hot (30°C) quadrants was manufactured using a design similar to that described by Arenas *et al*., (2017). *Luciferase(RNAi)* spent most of their time in the cold quadrants and avoided hot temperature quadrants (Supplementary Fig. S4), as expected from findings by Arenas *et al*., (2017). No significant difference was observed in the time spent in cold and hot quadrants between *Smed-TRPM-c(RNAi)* and control groups (Supplementary Fig. S4). This result supports the notion that *Smed-TRPM-c* is directly required for sperm development, and that spermatogenesis defects in *Smed-TRPM-c(RNAi)* animals are not indirect outcomes from compromised neuronal functions.

### *Smed-TRPM-c* supports development and/or maintenance of *nanos*(+) presumptive germline stem cells in asexual planarians

**Fig. 5.**
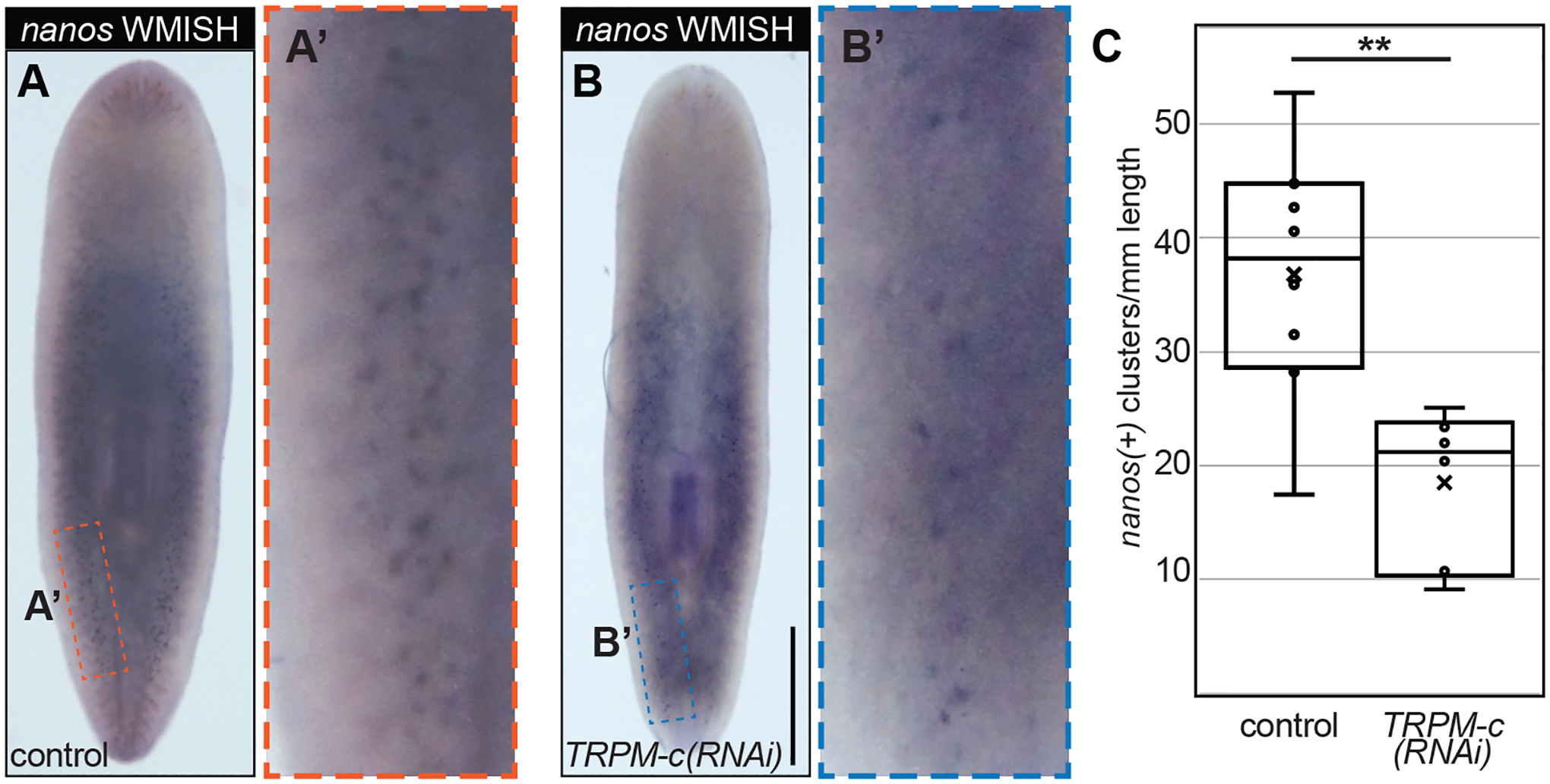
*Smed-TRPM-c* is required for maintenance of presumptive germline stem cells in asexual planarians. **(A,B)** Distribution of *nanos(+)* germline stem cell clusters in control *Luciferase(RNAi)* (A) and *Smed-TRPM-c(RNAi)* (B) asexual planarians are displayed in bright field microscopy images of samples subjected to whole-mount *in situ* hybridization. Insets in **(A’ and B’)** show 5-fold magnified view. Scale bar, 1 mm. **(C)** Box and whisker plot displaying quantitative analysis of *nanos(+)* cluster number in relation to planarian body length observed in *Luciferase(RNAi)* and *Smed-TRPM-c(RNAi)* planarians. Dots represent individual data points and “x” marks the mean. Statistical significance (Student’s *t*-test, *p* < 0.01) is indicated by two asterisks.

The phenotype observed from RNAi analyses in sexual planarians indicated that *Smed-TRPM-c* supports early stages of spermatogenesis. To further investigate this hypothesis, expression of *Smed-TRPM-c* was disrupted in asexual planarians. An asexual strain of *S. mediterranea* that reproduces exclusively through fission and regeneration contains a chromosomal translocation assumed to impede sexual maturation (Newmark and Sanchez Alvarado, 2002; Newmark *et al*., 2008). However, planarians in this asexual strain still contain presumptive germline stem cells located in the dorsolateral position equivalent to where testes develop in sexual planarians (Wang *et al*., 2007). Groups of asexual *S. mediterranea* were subjected to six-weeks of *Luciferase* or *Smed-TRPM-c* dsRNA feedings. Upon completion of dsRNA feedings, the presence of presumptive germline stem cell clusters was visualized using *nanos(+)* marker expression by WMISH. At the end of the 6-week RNAi treatment clusters of *nanos(+)* cells were quantified in both *Luciferase(RNAi)* control planarians (Fig. 5 A–A’) and *Smed-TRPM-c(RNAi)* planarians (Fig. 5 B–B’). Calculating the abundance of *nanos(+)* clusters per millimeter of animal length revealed a decrease in the number of *nanos(+)* clusters in *Smed-TRPM-c* knockdowns (Fig. 5C; see Supplementary Fig. S5 for individual *nanos(+)* cluster counts plotted against animal length). Statistical analysis using two-tailed Student’s *t*-test indicated that the difference in number of clusters per millimeter of animal length between *Smed-TRPM-c(RNAi)* and control planarians is significant (p < 0.01). This reduction in relative number of *nanos(+)* clusters corroborates with the loss of testis lobes observed in *Smed-TRPM-c(RNAi)* sexual planarians (Figs. 2 B,C,H and 4 B,C). Given these results, we propose that *Smed-TRPM-c* contributes to spermatogenesis through regulation of establishment, maintenance, and/or expansion of germline stem cells.

### Smed-TRPM-c has domains present in mammalian TRPMs and is predicted to form a homotetrameric transmembrane channel

**Fig. 6.**
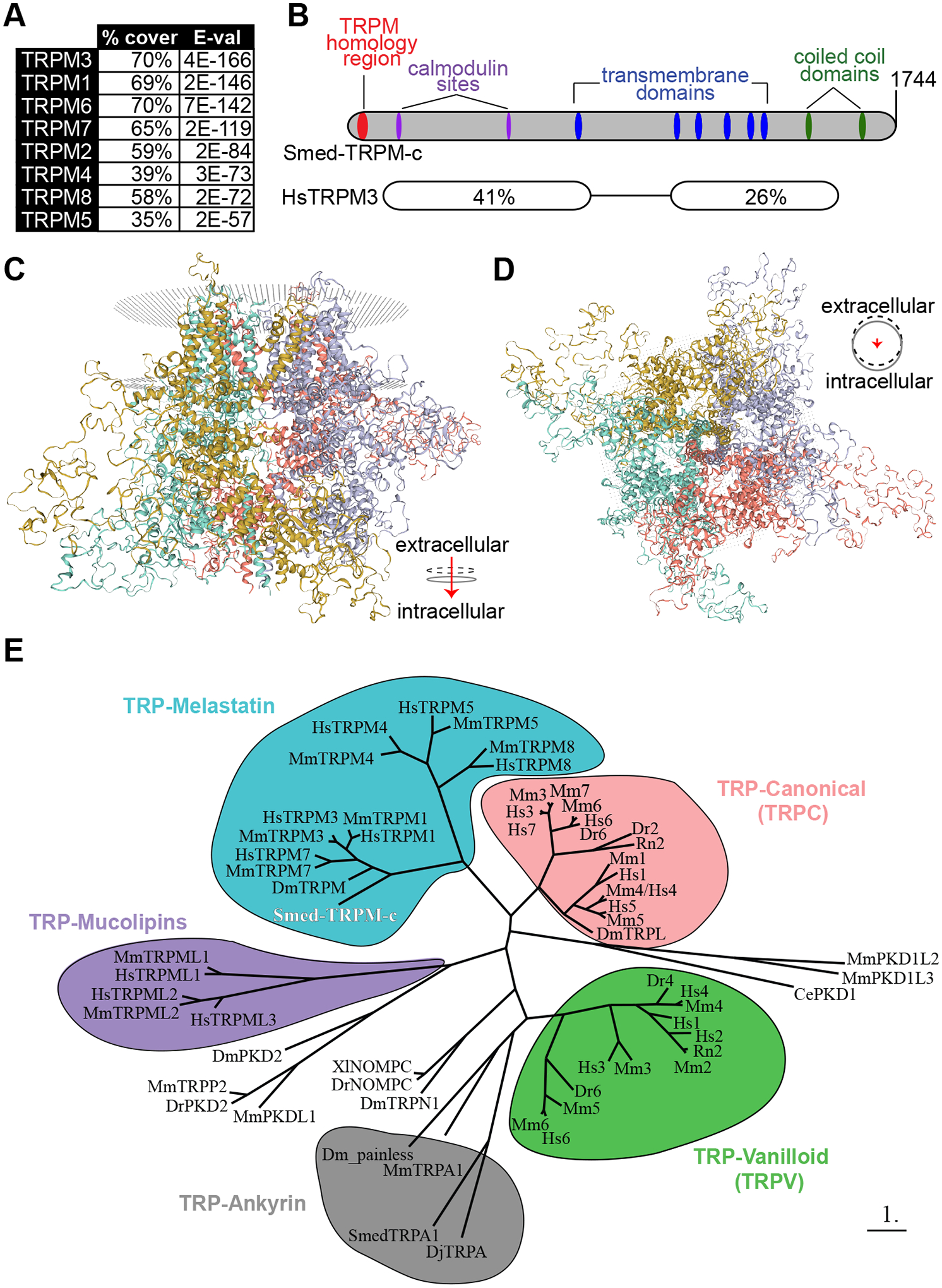
Predicted protein structure and phylogenetic analysis of Smed-TRPM-c. **(A)** The top eight human matches in BLASTP analysis against Smed-TRPM-c are TRPM proteins. **(B)** Architecture of Smed-TRPM-c illustrated using a bar diagram that includes predictions of transmembrane (blue) and coiled coil (green) domains identified by SMART (Simple Modular Architecture Research Tool; Letunic *et al*., 2021), as well as a TRP-Melastatin (TRPM) specific homology region (Grimm *et al*., 2003; red), and two calmodulin binding sites identified by the Calmodulin Target Database (http://calcium.uhnres.utoronto.ca/ctdb/ctdb/home.html; magenta). Regions of sequence conservation identified by pairwise BLASTP between Smed-TRPM-c and human TRPM3 (isoform 1; NP_066003.3), including percent identity and corresponding E-values. **(C,D)** The predicted quaternary structure of Smed-TRPM-c as a transmembrane homotetramer according to SWISS-MODEL structural modeling (Waterhouse *et al*., 2018; Bertoni *et al*., 2017) based on homology to mouse TRPM7 (Duan *et al*., 2018). The complex of four subunits is shown in its frontal view (C) and intra to extra cellular view (D). Each of four subunits are shown with different color and the phospholipid bilayer is represented by gray dots. **(E)** Phylogenetic tree using maximum-likelihood principle depicts closer association of Smed-TRPM-c (red bold font) with TRPM from *Drosophila melanogaster* (Dm) and mammalian TRPM3/7/1 orthologs, than with other characterized TRPs. Groups of TRPs belonging to the melastatin (blue), canonical (pink), vanilloid (green), ankyrin (gray), and mucolipins (purple) families are grouped in bubbles. Abbreviated names showing only first letter of genus and species names (e.g., “Hs” for *Homo sapiens*) were utilized for TRPC and TRPV genes. Scale bar represents 1 substitution per amino acid position.

BLASTP analysis of the protein sequence coded in the longest identified open reading frame (5,235 nts; GenBank: PQ273721.1) identified TRPM3 as the closest human homolog to this planarian gene (Fig. 6A; E-value = 4E-166), which corroborates with the analysis of reference cDNA contigs for *Smed-TRPM-c* deposited on PlanMine (Supplementary Table S1). Smed-TRPM-c contains long regions of conserved sequence that include several predicted structural features present in mammalian TRPMs (Fig. 6B). These structural features include the N-terminal TRPM Homology Region (MHR) that is characteristic of members of the TRPM family (Grimm *et al*., 2003; Wagner *et al*., 2008; Wang *et al*., 2018; Winkler *et al*., 2017; Supplementary Fig. S6), six transmembrane domains (Supplementary Fig. S7), two calmodulin binding sites found in human TRPM3 (Holakovska *et al*., 2012) and two C-terminal coiled-coil domains (Fig. 6B). The MHR of Smed-TRPM-c and human TRPMs are 26–37% identical (Supplementary Fig. S6). Additionally, the serine residue at the 1107 position of mouse TRPM7, which is important in modulation of channel activity in response to phosphatidylinositol 4,5-bisphosphate levels (Zhelay *et al*., 2018) is conserved in Smed-TRPM-c (Supplementary Fig. S7). Analysis of quaternary structure based on protein homology modeling (SWISS-MODEL; Waterhouse *et al*., 2018; Bertoni *et al*., 2017) predicted a homotetrameric transmembrane channel as the top formation for Smed-TRPM-c (Fig. 6 C,D). Each unit in the complex is predicted to contribute to the central pore of the channel (Fig. 6D). To infer the phylogenetic relationship between Smed-TRPM-c and TRPs characterized in other experimental models, a phylogeny tree based on maximum-likelihood (Dereeper *et al*., 2008) was generated (Fig. 6E). In this analysis, Smed-TRPM-c groups with a subset of TRPMs that includes mammalian TRPM1, 3, and 7, as well as TRPM from *Drosophila*, which are expressed in spermatogenic cells in mammals (Li *et al*., 2010) and mediate calcium influx during egg activation in *Drosophila* (Hu *et al*., 2019), respectively. Altogether, these analyses indicate that Smed-TRPM-c shares sequence and structural features that are typical of mammalian TRPMs and close sequence conservation with those expressed in germ cells of other animals.

Intracellular enzymatic domains are present in C-termini of a few mammalian TRPMs (*i.e*., TRPM2, TRPM6, and TRPM7), which is not characteristic of other TRP channels (reviewed by Scharenberg, 2005; Cabezas-Bratesco *et al*., 2015). Examination using bioinformatic domain recognition software and BLAST analyses did not identify a kinase domain homologous to those present in mammalian TRPM6 and TRPM7 amongst any of the planarian TRPMs. The closest sequence to match the kinase domain of human TRPM6 and TRPM7 in *S. mediterranea* is that of a eukaryotic elongation factor 2 kinase ortholog (data not shown). In contrast, a match for the NUDIX hydrolase domain present in the C-terminus of human TRPM2 was detected in C-termini of Smed-TRPM-a1 and Smed-TRPM-f, as well as in DjTRPMa from the planarian *D. japonica* (Supplementary Fig. S8). These findings support the notion that TRPM channel fusions with NUDIX hydrolase enzymatic domains predate the cnidarian-bilaterian split, as hypothesized from detection of this domain in TRPM2 from the cnidarian *Nematostella vectensis* (Kühn *et al*., 2016). Interestingly, all three of the planarian proteins that possess homology to the hydrolase domain (Smed-TRPM-a1, DjTRPMa, and Smed-TRPM-f) are preferentially expressed in neurons (Fig. 1 and Supplementary Table S1; Inoue *et al*., 2014).

## Discussion

Our research revealed 21 potential TRPM family members in the planarian *S. mediterranea*. Expression of two of these was detected in testes, while some of the others were expressed in the gut, head tip, and subsets of neurons (Fig. 1). *Smed-TRPM-c* expression is enriched in testes (Figs. 1, 3, and Supplementary Fig. S1), which parallels expression of *TRPM3* in mouse testis (Jang *et al*., 2012) and in rat spermatogenic cells (Li *et al*., 2010). Expression in the ovaries was only observed in a subset of animals possibly due to incomplete development of the ovaries, which occurs towards the end of sexual maturation. *Smed-TRPM-c* is required to maintain normal distribution and development of active testes in the dorsolateral anatomy of sexual planarians (Figs. 2, 4) and presumptive germ cell clusters in asexual planarians (Fig. 5; Supplementary Fig. S5). We hypothesize that *Smed-TRPM-c* contributes to spermatogenesis via cell-autonomous mechanisms based on detection of expression of this gene in the outer layer of germ cells in testis lobes, which is where spermatogonial stem cells and spermatogonia reside (Fig. 3 D–D’, Supplementary Fig. S1). We also hypothesize that *Smed-TRPM-c* works to regulate sperm development in the earliest steps of spermatogenesis (*i.e*., establishment, proliferation, and/or maintenance of germline stem cells), based on the observation that presumptive spermatogonial stem cell clusters are less abundant upon reduction of *Smed-TRPM-c* expression in asexual *S. mediterranea* (Fig. 5; Supplementary Fig. S5). These results place Smed-TRPM-c as a sensor of either cellular or environmental cues that drive sperm development in *S. mediterranea*.

Members of the TRPM channel family are known to be activated by cold (TRPM8; McKemy *et al*., 2002) and hot temperatures (TRPM3; Vriens *et al*., 2011), as well as by chemical ligands such as the steroid hormone pregnenolone sulphate and spermine (reviewed by Huang *et al*., 2020). The stimuli that activate Smed-TRPM-c remain unknown. However, our preferred hypothesis is that Smed-TRPM-c is responsive to temperature and regulates cell-autonomous mechanisms in the male germline. This is based on three indirect observations: 1) A member of the TRPM family that displays preferential expression during spermatogenesis in mammals protects germ cells from damages induced by cold-shock (TRPM8; Borowiec *et al*., 2016); 2) Smed-TRPM-c shares the highest sequence conservation with TRPM3 amongst mammalian orthologs, and mammalian TRPM3 is responsive to heat; 3) sexual reproduction and development in planarians are heavily regulated by temperature (see below); and 4) we didn’t detect any sperm development defects upon knockdown of orthologs of genes involved in pregnenolone sulphate metabolism (data not shown).

Planarians inhabit freshwater ecosystems all over the world, with Antarctica and islands where colonization has not occurred being possible exceptions (Vila-Farré and Rink, 2018). Most species in the genus *Schmidtea* are primarily distributed in northern Europe, while *S. mediterranea* has been mainly reported to inhabit southern European regions such as Tunisia, Italy, and Catalonia (Lázaro *et al*., 2011). Exclusively fissiparous populations (*i.e*., asexually reproducing through fission and regeneration) of *S. mediterranea* and other planarian species are not uncommon. However, sexual reproduction by hermaphroditism is the predominant and ancestral mechanism for reproduction in planarians. Development, size, and activity of planarian reproductive structures in the wild follow seasonal patterns, and some planarian species even switch between sexual and fissiparous reproductive strategies (Dahm, 1958; Ball and Reynoldson, 1981; Nodono and Matsumoto, 2022). Temperature has long-been an abiotic factor of interest in modulation of planarian reproductive strategies. For example, *S. mediterranea* testes, ovaries, and the copulatory apparatus shrink and eventually disappear in water temperatures above 20°C in (Harrath *et al*., 2004). However, the environmental and molecular factors that regulate this sexual maturation and reproduction in the wild remain to be uncovered.

Some evidence suggests that temperature works to tune sexual maturity in planarians through independent tissue-specific pathways, rather than through a master regulator. For example, *Schmidtea (Dugesia) lugubris* exhibits regressed testes at temperatures below 5°C, but these grow and become fertile when transferred to 10°C (Reynoldson *et al*., 1965). In contrast, *S. lugubris* ovaries are populated with oocytes even at temperatures below 5°C, indicating that testes and ovaries do not follow the same temperature restrictions. In another example, *Polycelis tenuis* produces capsules below 5°C, indicating that vitellaria are present and functional, but these capsules are sterile. Given that gametes are not required for capsule production in planarians (Steiner *et al*., 2016), the observation that *P. tenuis* produces capsules at low temperatures indicates that vitellaria are fully developed and functional, while their sterility suggests that sperm, ova, or both are absent or dysfunctional. In contrast, the planarian species *Dendrocoelum lacteum* produces fertile capsules at temperatures as low as 1°C to 5°C, indicating that functional oocytes, sperm, and vitellaria are present and functional in *D. lacteum* at these low temperatures. The stimulus (or stimuli) that activates Smed-TRPM-c remains to be found. However, *Smed-TRPM-c* does not seem to function as a “master switch” that modulates reproductive maturation across tissues. Instead, *Smed-TRPM-c* seems to serve primarily in the testis and promote specification, proliferation, and/or maintenance of spermatogonial germline stem cells. Tissue-specific functions of sensory receptors like *Smed-TRPM-c* provide a mechanism by which planarians can evolve reproductive system strategies in ways that maximize fitness in the different freshwater ecosystems that they inhabit.

## Materials and Methods

### Planarian cultures

A laboratory line of sexual *S. mediterranea* (Zayas *et al*., 2005) was used for all experiments except for those indicated as specifically using asexual animals, in which case specimens from the CIW4 clonal laboratory strain were used (Newmark and Sánchez Alvarado, 2000). Asexual animals were maintained in 1X Montjuïc salts at 21°C. Sexual animals were maintained in 0.75X Montjuïc salts at 18°C. Planarians were maintained in the dark except during weekly or biweekly feedings with beef calf liver, which were done on bench tops at room temperature. Laboratory colonies of *S. mediterranea* sexual strain were maintained and amplified mainly through transverse amputation and regeneration. Therefore, the majority of sexual animals used in this study originated from regenerated fragments. Animals of comparable size were chosen at the start of every experiment, checked for absence of gonopore on the ventral posterior (when juvenile stage was desired), and starved for at least one week prior to experimentation or fixation.

### Identification of TRPM homologs and generation of riboprobes for whole-mount *in situ* hybridization

TRPM homologs in *S. mediterranea* were identified from transcriptomes of sexual (dd_Smes_v1 prefix) and asexual (dd_Smed_v6 prefix) strains deposited in PlanMine (Rozanski *et al*., 2019) using human TRPM3 (NCBI GenBank ID NP_066003.3) as input in TBLASTN searches. Redundant records were identified by pairwise BLASTN comparisons and removed. GeneArt Strings DNA fragments (ThermoFisher, Waltham, MA) were synthesized for each *S. mediterranea* TRPM homolog to contain ~500 bps of reference contig sequence flanked by SP6 (sense) and T3 (antisense) promoter sequences (Supplementary File 1). These custom-ordered DNA fragments were amplified by PCR using primers corresponding to SP6 and T3 promoter sequences that included an additional T7 promoter sequence at their 5’-ends (5′-GAATTTAATACGACTCACTATAGGGCGATTTAGGTGACACTATAGAAGAGAAC-3′ and 5′-GAATTTAATACGACTCACTATAGGGCGAATTAACCCTCACTAAAGGGAAC-3′, respectively) as described in Counts *et al*., (2017). PCR fragments were purified using DNA Clean & Concentrator-5 kits as per the manufacturer (Zymo Research, Irvine, CA), eluted in 20 μL or RNase-free water, and used as templates for synthesis of riboprobes labeled with Digoxigenin-11-UTP (Roche, distributed by Millipore Sigma, Burlington, MA) using T3 RNA polymerase (Promega, Madison, WI).

### Cloning of *Smed-TRPM-c* cDNA

Full-length mRNA sequence for *Smed-TRPM-c* was predicted from matching contigs (dd_Smed_v6_11377_0_1, dd_Smes_v1_41098_1_4, uc_Smed_v2_27847, others) identified in in PlanMine (Rozanski *et al*., 2019) and primers for 5’ and 3’ RACE [5’-AGGAAGCAATGATTTACCGGGAGAAAG-3’ and 5’-CAAGTGGTCAGTAGAGCGCATTGACTAC-3’] were designed based on this prediction. Full-length *Smed-TRPM-c* ORF was amplified using Long PCR Master Mix (Promega, Madison, WI) from sexual *S. mediterranea* cDNA synthesized using oligo(dT) and N_6_ primers. The oligo primers used for amplification of *Smed-TRPM-c* ORF were 5’-GTCACAGTGGCATTGTAGCCAATCCCTC-3’ and 5’- ATTGGCGAGTAAATCGCTTTGCATTGC-3’. The forward primer is position immediately upstream from the first 19 nucleotides of the ORF (5’-ATGAAAAAATCTAAAAAAA-3’) according to records on PlanMine. Primers were not designed to include this region due to rich A/T content. Amplicons were ligated into the vector pGEM-T (Promega, Madison, WI) and verified by Sanger sequencing.

### Bioinformatic analysis of protein structure

The transmembrane and coiled coil domains of the predicted *Smed-TRPM-c* full-length ORF were identified using the Normal SMART program (Letunic *et al*., 2021). The starting motif of the TRPM Homology Region (MHR) was derived from Grimm *et al*., (2003). The putative calmodulin binding sites of Smed-TRPM-c were identified with the Calmodulin Target Database (http://calcium.uhnres.utoronto.ca/ctdb/ctdb/home.html). N-terminus TRPM homology region analysis was performed by alignment of TRPM Homology Region of different TRPM channels (Grimm *et al*., 2003) using ClustalW 2.1 (Thompson *et al*., 1994). The multiple alignment was then input into BoxShade (https://github.com/mdbaron42/pyBoxshade/releases) using the default settings. Predictive models of three-dimensional Smed-TRPM-c structure were obtained using SWISS-MODEL structural modeling (https://swissmodel.expasy.org/; Waterhouse *et al*., 2018; Bertoni *et al*., 2017) based on homology to mouse TRPM7 (Duan *et al*., 2018) and using default settings.

### Smed-TRPM-c phylogenetic analysis

TRP protein sequences were obtained from analyses published in Inoue *et al*., (2014) and Arenas *et al*., (2017). The predicted amino acid sequence of Smed-TRPM-c was aligned with those of TRP proteins from these studies using ClustalW and used as input to generate a phylogenetic tree using PhyML and TreeDyn in phylogeny.fr (Dereeper *et al*., 2008). Default settings were used across programs.

### Whole-mount *in situ* hybridization

Whole-mount *in situ* hybridization was performed as described by Pearson *et al*., (2009) with modifications in fixation steps following King and Newmark (2013). Large sexually mature animals (~1.0 cm or larger) were used, therefore the N-acetylcysteine (NAC) treatment, as well as the initial fixation step and the Proteinase K treatment were prolonged. Planarians were placed in a solution of 10% NAC in PBS for 11 minutes with gentle agitation, then fixed in 4% formaldehyde in PBS containing 0.3% Triton X-100 (PBSTx) and rocked for 1 hour at 4°C. The animals were then gradually dehydrated in methanol and kept at −20°C overnight. The following day, the samples were gradually rehydrated in PBSTx and bleached in formamide bleaching solution (King and Newmark, 2013) for 2 hours under a bright light. After bleaching, the samples were rinsed in PBSTx and placed in a Proteinase K solution containing 10μg/ml of Proteinase K (ThermoFisher, Waltham, MA) in PBSTx with 0.1% (w/v) SDS for 12 minutes with gentle agitation. The samples were post-fixed for 10 minutes with 4% formaldehyde in PBSTx. The steps for hybridization and post-hybridization washes were performed as indicated by Pearson *et al*., (2009). For colorimetric *in situ* hybridization, samples were incubated in blocking solution containing 5% horse serum in TNTx (consisting of 0.1M Tris pH 7.5, 0.15M NaCl, and 0.3% Triton X-100) for at least 2 hours. Samples subjected to colorimetric signal detection were incubated in anti-Digoxigenin-AP antibody solution (1:4000 dilution; Roche Diagnostics, Mannheim, Germany) overnight while rocking at 4°C. The post-antibody incubation washes with TNTx and signal development were performed as in Pearson *et al*., (2009) for colorimetric signal detection. After development, the samples were mounted in 80% glycerol/20% PBS and imaged using a Zeiss V.16 SteREO microscope equipped with a Canon EOS Rebel T3 camera. For fluorescent *in situ* hybridization, samples were incubated in blocking solution containing 5% horse serum and 1% Western Blocking Reagent (Roche Diagnostics, Mannheim, Germany) in TNTx for at least 2 hours. Samples used in FISH analyses were incubated in anti-DIG-POD antibody solution (1:2000 dilution; Roche Diagnostics, Mannheim, Germany) rocking overnight at 4°C. The FISH samples were developed using FAM tyramide solution as described by King and Newmark (2013), washed, and mounted in 80% glycerol/20% PBS and imaged using a Nikon C2+ confocal microscope with NIS Elements C software. Initial observations of distribution of expression of TRPM homologs were performed using riboprobes generated from GeneArt DNA fragments (Supplementary File S1), and *Smed-TRPM-c* expression was validated in colorimetric and FISH analyses using riboprobes generated from full-length ORF clones. Modifications of WMISH for double-FISH were performed as per King and Newmark (2013).

### Disruption of *Smed-TRPM-c* expression by RNA-interference (RNAi)

Templates for *in vitro* transcription of dsRNA generated by PCR from GeneArt fragments (Supplementary File S1) using the aforementioned primers (see “generation of riboprobes for whole-mount *in situ* hybridization, above) or from cDNA cloned into pGEM-T (Promega, Madison, WI) using the following primers: 5’-GAATTTAATACGACTCACTATAGGGCGCCAAGCTATTTAGGTGACACTATAGAATACTC-3’ and 5’- GAATTAATTAACCCTCACTAAAGGGAGAATTTAATACGACTCACTATAGGGCGAATTGG-3’). PCR products were purified using DNA Clean & Concentrator-5 columns (Zymo Research, Irvine, CA), eluted in 20μl of RNase-free water and used as templates for *in vitro* transcription. DsRNA was synthesized using T7 RNA polymerase as described by Rouhana *et al*., (2013).

Depending on the experiment, groups of either 6 juvenile sexual planarians (lacking gonopores), mature sexual planarians actively laying capsules, or 10 asexual planarians 0.5–0.7 mm length, were fed to satiation with a liver solution containing approximately 100 ng/μL of gene-specific dsRNA twice per week for at least 6 weeks. As a negative control, one group of planarians was fed firefly *Luciferase* dsRNA which is not expressed in the planarian genome and has no effect on their sexual development nor behavior. To assess RNAi efficacy in real-time, some experiments included one group of planarians fed with *Smed-pc2* dsRNA which is required for testes maintenance and normal planarian behavior (Reddien *et al*., 2005; Collins *et al*., 2010).

### Analysis of testis distribution and sperm development

A week following completion of the last RNAi feeding, planarian testis distribution and anatomy were assessed by FISH and/or DAPI staining. The samples were fixed and bleached as described above for *in situ* hybridization and processed for FISH, or (without methanol dehydration and rehydration steps) for analysis by DAPI only. After bleaching, DAPI-only samples were washed in PBSTx twice and then incubated in DAPI (1:1000 dilution of 1mg/ml DAPI stock solution in PBSTx; ACROS Organics, Morris, NJ) overnight while rocking at 4°C. After incubating overnight, the samples were washed 4 times with PBSTx and then mounted on slides with 4:1 glycerol:PBS and imaged under UV light with a Zeiss V.16 SteREO microscope equipped with a Canon EOS Rebel T3 camera (for low magnification) or a Nikon C2+ confocal microscope using a 10X or 20X objective and running NIS Elements C software (for high magnification).

### Analysis of ovarian anatomy, oocyte development, and egg capsule deposition

To assess possible disruptions to oogenesis, the number of oocytes per ovary of *Smed-TRPM-c* knockdown planarians were compared to *Luciferase* knockdown planarians. Juvenile sexual planarians fed gene-specific dsRNA for 6 weeks were stained with DAPI a week following the conclusion of the RNAi feeding regimen. The number of oocytes were counted using Z-stacks on a Nikon C2+ confocal microscope with NIS Elements C software. The ovaries of five *Luciferase(RNAi)* and five *Smed-TRPM-c(RNAi)* planarians were examined. Each count of oocytes was plotted as an individual point in a box and whisker plot. Statistical analysis was performed using unpaired two-tailed Student’s *t*-test. To assess egg capsule deposition, groups of 6 sexually mature planarians (1.0 – 1.5 cm in length) were used per group. The animals were fed dsRNA as described above. The cumulative number of egg capsules deposited was recorded after each dsRNA feeding.

### Analysis of presumptive germline stem cell distribution in asexual planarians

To assess distribution of presumptive germline stem cells in asexual planarians, groups of asexual *S. mediterranea* were subjected to dsRNA feedings for six weeks, fixed a week following the last feeding, and processed for colorimetric *in situ* hybridization using a *nanos* riboprobe (Wang *et al*., 2007). After *in situ* hybridization, each animal was imaged with a Zeiss V.16 SteREO microscope equipped with a Canon EOS Rebel T3 camera and the length of each animal was determined by drawing a 3-point arc from the center of the head to the pharynx and to the center of the tail of each planarian image using Adobe Illustrator. The images were each assigned a random number and subjected to triple-blind analysis. The number of *nanos(+)* clusters were counted by three individuals, averaged, and plotted against the length of the animal. Statistical analysis was completed using two-tailed unequal variance Student’s *t*-test on the ratios between the number of *nanos* clusters and the length of the animal.

### Thermotaxis assay

Thermotactic behavior of asexual planarians was tested with an aluminum temperature plate based on the design used by Arenas *et al*., (2017) (Supplementary Fig. S4). The aluminum temperature plate was controlled by a simple variable 12-volt power supply. The power supply was regulated by two temperature controllers (Elitech, Milpitas, CA). The temperature controllers fed four Peltier plates in diagonal quadrants. Each pair of Peltier plates were either powered by positive or negative 12-volt direct current. Positive current produced warmer above-ambient temperatures while the negative current produced below-ambient temperatures.

To assemble the apparatus, thermal silver paste was used to adhere the anodized aluminum plate to the Peltier plates. The aluminum plate was treated with a hydrophobic waterproofing spray while leaving an uncoated circle of diameter 8.6 cm in the center. Two temperature sensors were adhered to the perimeter of two adjacent quadrants of the untreated circle. The heat sink was adhered to the bottom of the Peltier plates using the same thermal paste. A small computer fan was attached to the bottom of the heat sink to assist with heat dispersion.

The untreated circle was filled with Montjuïc salts to a height of approximately 3 mm, forming a bubble of water for the samples to move through. Groups of 10 asexual planarians were subjected to six weeks of RNAi treatment and then starved for one week prior to experimentation. Four *Luciferase(RNAi)* planarians and five *Smed-TRPM-c(RNAi)* planarians were fed pureed beef liver mixed with colored chalk shavings (Hagoromo Co., Ltd., Kasugai, Japan) as per Hattori *et al*., (2018) prior to being placed on the temperature plate to increase the visibility of the planarians during the assay. The nine planarians were placed in the center of the circle together and their movements were recorded for 10 minutes. Heat map images were taken with a thermal camera (Hti-Xintai, Dongguan, Guangdong, China) at five-minute intervals. The percentage of time spent in cold quadrants for each individual planarian was calculated from the video recording and plotted on Excel. Statistical analysis was performed using two-tailed unequal variance Student’s *t*-test.

## Supplementary Material

Supplementary Material
