# Supplementary material for: Melastatin family Transient Receptor Potential channels support spermatogenesis in planarian flatworms
Source: Int J Dev Biol. Author manuscript; Available in PMC 2025 Sep 10. (PMC12421698; doi:10.1387/ijdb.240180lr)
Supplement: Supplementary Material [file NIHMS2109286-supplement-Supplementary_Material.pdf]

THE INTERNATIONAL JOURNAL OF DEVELOPMENTAL BIOLOGY
www.intjdevbiol.com

Supplementary Material

corresponding to:

Melastatin family Transient Receptor Potential channels support spermatogenesis in planarian flatworms

HALEY NICOLE CURRY, ROGER HUYNH, LABIB ROUHANA

Full Text for this paper is available at: https://doi.org/10.1387/ijdb.240180lr

Supplementary File S1. Sequence of GeneArt Strings Gene Synthesis constructs used for initial analyses of *S. mediterranea* TRPM homologs. Sequence of templates used to generate amplicons for *in vitro* transcription of DIG-labeled riboprobes and dsRNA against *S. mediterranea* TRPMs. Partial *Smed-TRPM* paralog reference contig sequences (lower case font) are flanked by sense SP6 and antisense T3 RNA Polymerase promoter sequences in their 3'- and 5'- ends (capitalized font), respectively.

>Smed-TRPM-a1
GCGATTTAGGTGACACTATAGAAGAGAACcaatttatgaatttgaaaatttatccccggacagtctcttc
gattacaaattagaggaaacgttgaaggtgtcttcgaatttcttaagctaccttgataattgcaacttgc
ttcactatgccgctttgtatagagcaactagaatagtcagggtacttttaagttcacagtttaactaccc
agttgattcaatgaccaaggaaggaataacgccacttcacatttctgcagccgtgggagatttctatact
gtccgagaacttctaacgcatggagcaaataagaatttgttggatcaaaaaggaagaatagctttacatt
atgcagtatgttccaatgtcgagacttgcatcatcctcacaagtgatgggaattatgatctacaacttca
agatgagaatgaaaagtcaccactgcaatacggtaaaatggaagacaagccatttattgccaagtattta
gaaacattagataaaaagaaactaaacctatggatacctcagaatatttgccagtttgaatgttattcca
tcattccaagaaaggaaagccttattcagtgcgaatgtgggcaaaatgttgccgatcatcattcagtttt
cgaaaaatatgatttcgttgacacggaaaatgttgaaaaacccacaaatttctttggcgaaattGTTCCC
TTTAGTGAGGGTTAATTCGCCCT

>Smed-TRPM-a2
GCGATTTAGGTGACACTATAGAAGAGAACcaagacgatgctttcaaccagctaaatattccacaactaaa
catttcgatttcaataaatagacatgatgttgccagggagaaaatctttacggaaggcaagaaatggaaa
acagaagatttaatgcccttggcatcaaaatgtgtgtctgacaacaaagttgaattcataaagatttta
ttgaaaagggatttttctagcgaaaataataacagttgagtatttggaatacctttatacgtctgacgt
tttcaatggatacaaatcacttcgaatactccatcaagttttgagattgacgaaatttaagaaaagtaac
aatcgagtatctctatatgacattggggcagtcctaaaatatatgcttggtgacttttactttcccctct
atttgaaaagctcattccagtcaactttaaatgaaaatttagagctgactagaccctatcgagagttatt
tatatggagtttgctaacacaaagaatggaacttgcagaacttctttggactttagaaactgaatcaatt
gcagctgcattgttcgctgcaaatttgttgcggtgcatgataaatcttactgatgttatttcagatcgag
aagaccttgataaatgggcaggaatcttcgaaaataaagctgaaggggttgtggaagaatgctttaaaga
gaaccccgaatatacacGTTCCCTTTAGTGAGGGTTAATTCGCCCT

>Smed-TRPM-b
GCGATTTAGGTGACACTATAGAAGAGAACtgatagcattatttgcctacggaacattttctatgtcttta
ttgtatccgaatataaacatcacgagtccattgatgagaaatatttttataatccttattggcaattgt
ttggtcaagttgacacttcttatatggaaggcagtcagtgtctttttggatattgtgcaactccaaatgc
aatagtccaagttgctaatgctttctttttattattcaccaacgttttactgctcagtcttcttattgct
atgtttggagtcacatttatgaaggtagaagagaactcgaatcgtgtttgggcgtttactcgatatcgga
tgattgaagaattctactaccgaccaattttgccacccccttttcaatgtcttcgaagttttctataattt
tttcaagttgaatatctgcaaaaattctaacaatggccggttatcggttgatgcattcattgcgaatttg
atccacgaagaagaccttgaatgttccggttttgaacaacagtgcatggagacatatttaataaccaacc
aattgttagatctttcagctcatgatgctagtaaagcatgtttcaaatctgatgattccataacaaatga
acagtctaatgttgctatagaggcccacgtaaaagctcttgttggttctattgcagaaataaaagatgaa
ttaaaaGTTCCCTTTAGTGAGGGTTAATTCGCCCT

>Smed-TRPM-c
GCGATTTAGGTGACACTATAGAAGAGAACccttcagtgcaccaataacaacctcaacgacctaaacatca
actacaactgccaaagtgcagctttattggaaaaaataagtgaatttgaaaataaattgattaatactcc
gggtcacatatcatttttcaattgtaaaaataatacaaattcagtatgtgacggctcagagaaggctgaa
gataataataaaaataattcaatgttaaatgctgaacggaaaaatgtcagtaaacttatttctgaaagtg
ctcactgtgatagtccttttgagaaaactgttagcagaaaccacattttcggatttccgaactacaagca
gaaaagtattccgaaatcataccaatccacttatccgattaccgacaagtacacgatacccctccgcaga
gattatacaacgatttgtgacgacatcattatatcagattggatatcgactcgagaaactcctccggcca
gtccaacctccagcatgattgatttgagtcatccaggaaaatcgaaaaggtcaaatcgtctcatgaaatc
gaaagcgaaacgcaataaactcttaagtaataaagagataattatcggtgacaaagaagaagagttgcga

caagccgagaaaatagaacgagaagaaatgaccagtgttatcgtccaaaggcttcgacaattgtctcata
agcaaacttattcatctaccgGTTCCCTTTAGTGAGGGTTAATTCGCCCT

>Smed-TRPM-d
GCGATTTAGGTGACACTATAGAAGAGAACcaaggagcttattttcctcaaagagattttagtctgaatac
tctaccgaatataatggactacccgtactgggctatatttggggagattacattgaatcaattggtttat
ttgccttggtgtgatgacaaaacaacaaattataatatttcaaatgaatgccctgaactcaactattttg
ttccggttttgacagctatatatatttttattgcagctatattattattaaatatcctgactgcagtttt
caactacatttttcaaagaattcacagaaattcgatagaaatctggaaattcacaatgttttctttggtt
atggaatattatacgaaaccgctttttccgctgcctttttctatatttgctcacgcgtttgactttttct
actcactctgtggaaaccaatgtgaaaaaactgaagaaaatgaaaaaaatctcgacaccttgagcagaga
ttctcaaattactttttctcctccgaaaaaaatgaaatctttcaactttagaaaactagcaataccatta
gaatcagaaattgataaattagttaaatatttcgaaaacaactcccaaaaatgtgtggtaaaagaacttg
ttgaagaaaaagagcaattaattgaatcgggaatgtctcgactggaatctcgattaaaggcaatgtctac
gattataaaatctattgagGTTCCCTTTAGTGAGGGTTAATTCGCCCT

>Smed-TRPM-e
GCGATTTAGGTGACACTATAGAAGAGAACggataaatttgtcgagatattcaagtcaagatccgatagaa
tttatggtcgcataaagtattggtggacctttaatcactggaacaaggttgatatgattattatagtttt
tgcaatagttgcgatggtattgcgataccaactggccatcaatggtgtctacacagacaaagttgttatg
tttcgctggtcaaaaaccatatatagtctaatagtcggatcgttttacttgaaaattttgcggctactgg
catgtcacaaaaaattgggtccgaaaatttttatgatgtcaaaaattattaaagagcttctgatattttc
aacgatcatattaatctttcttttgtcatatggcgttacaagtcaggctggatattatccacaaagagat
ttttcatggattgcactatcaaatattttagattacccttactatacgatgttcggggttttaaatttgg
ggaacactgtctatttcacttggtgcaacgccagtacaaattacgaggtatctaatgaatgtcctgagaa
taatttttttaattccaatcttatttgcgggatatgtgataattatcaacattttattattgaatattctt
atagccacatccagttacatttgtcaagatattcatcgaaattcaattgtcatatggaaatacagcatgt
GTTCCCTTTAGTGAGGGTTAATTCGCCCT

>Smed-TRPM-f
GCGATTTAGGTGACACTATAGAAGAGAACaaccccagaagtaaatatacaaaccattctacaaatcaaat
gggaattatatcatcctaccctcgttattagcatttttggtggagatttcgaaaagaaacgaaaactaaa
aatgattattaaaaaaggcctctggaaagcagcagaaagtgctgggtgttggattataacagaaggttta
aatatgggaataatgaaacttgcaggagaagctgttcgagattatactgacgcatatggaggcaataaaa
tggtagcgataggaatcgcaccatggaacgtagtggcaaagaatgacgttttagaaaattctctttacca
aggtacttatccagcaatttatcagtcggaagaagagtcggaaaccaatgatgacgggatggttaaggat
agaaataaagaaaagtcacttgacacgaatcattcgtacttcttcttgtatgattgtccagcgtcgtcga
aggaaatatcggaacctgatgtacaattcagatcacgatttgaacattcaatttcgttttggaaatctca
gacttctagctatgaaacgtcaaatgatacgagagttccgatttgtggattagtggttggtggagacatt
ctgactcttagacaagttttcaattcaataatcgaaaataaatgcccagtcgtagtatgccagggatcga
gtggagcagctgacgGTTCCCTTTAGTGAGGGTTAATTCGCCCT

>Smed-TRPM-g
GCGATTTAGGTGACACTATAGAAGAGAACgatgatggttatcggcttcaaatgtctaggaaaaaagttgc
ttattttcgacaagcccttgaaaataagattagtcaaaaactaggggagagagcagcaattcctgtagtt
ctgttaattgtagaaggtgggcatgaagtattggttgaaggaagtaaaagaataaaagaaaatgtcccat
taataatttgcgttggaacaggtcgagcttccaatattttaggagaagtttttcaagaagtttcaaattc
atcttggaagcgcgaaagaactcgttcaggggatacatcagtttttttccgaaaactatagaaaagcttta
aaaacaaaactcattgaacagaatatttgtgagctccatcaagttgatgaggatgtcaaatgcattgaag
aaatagttaaaaaggaatttcttattactctttttttatataaaatcacatcaagatttggattttgcaat

attgcactcattgattaaacggatggactttttgtgtgatcctcgatataatccgttgtcaagccaatta
tctctttgcctcatttggaatagaatcgacattgttaaagataagatctttttgggagatatttccctgt
caaacgctgaatggattgcgtttttcaaagagtcaattatgaaagggcgcattgattttattgaattatt
tttggaaaatgtttcGTTCCCTTTAGTGAGGGTTAATTCGCCCT

>Smed-TRPM-h
GCGATTTAGGTGACACTATAGAAGAGAACtggtccgaagattataatgatcagggacatgcttcgagata
ttttgttctttttgagcatatttgtcattttcgtgatcacttatggaatattggcacgatcgttgaagta
tccaaatagcgaattcaacacattacttttcaaagatattgtctacactcctttctttcaaacatttggt
gaattgatgttggaaaatttcgatgtggctggaccgtgtaatggaactgactgtatagtttcatctggaa
ctgtgttaataagttttgcagtctttttattaatgacaaatgttttgctgatcaatcttttaatcgcaat
gttcggctatactttcaatcaaattcaagaagactcggagaaattgtggtgcttcattagaatccagctg
gtgcaagagttttatggaaaacccatatttcctccaccactgaacatcttctaccacgcttttcgaatta
taagtttaccatgcaagtcacgttgtccaaaattaatatttgaacaagagaacaatttgatgttggagca
ttgtaaaccttctaaaagtttaaaacttaataatttcgagcagttttgtttgaatgagtctattttggaa
tctaataacaatacgtcggataacaaggataaatcgaataacgaaacggatttttttcggcaaagtcaatg
taaagcacgaggtacccgatGTTCCCTTTAGTGAGGGTTAATTCGCCCT

>Smed-TRPM-i
GCGATTTAGGTGACACTATAGAAGAGAACggagtcaggtcgagcagcagatttgatatgtgaaattctcg
aaacagataaaatgaatgttgaaatcgttgacaaatatgaggaacagctcagattgaccgatgacgataa
aatccgcattatagaaattctttctgatatatactctaactacaaagaaagaataactattcatgacctt
ctagatcaaaatgtgcacctggataaaaaaatacttgatgcaattatttctagtcgtgaagacagaaact
tagaaaagactctaaacttagcagttagatttaattgcgttgatattgctgatttcaaagtattccaatc
gtctagagcttccactctatctcaaaaaactttgggaatttattttaaagaaatgctaagcaagaatcag
catgagatgttacagttgtttttagtaaataatttcagcattgatgaatttgttcaagaaaacctccaag
atttatatatgacatctctacgatcaatcaaatgccgttgcacagaaatgatatctgatttgattttaag
agaggataaatcattcaaaattgacaatataaataatccgggtaaaaacctcctgaaagttatttacaaa
atcatctgtaatctaagtgacaacattttaaaattcgaatcttttaatttacgttcatctaattctcata
atgccaatcaaaattcttaccGTTCCCTTTAGTGAGGGTTAATTCGCCCT

>Smed-TRPM-j
GCGATTTAGGTGACACTATAGAAGAGAACcgtcaaacgaaactaatcaaacagattcttttacttcttct
cccaaattggaattcaacccctgtgatatgtttggcacaattacatttgaaggaaatgacaacctttcat
cggatcaacatcccaattttatgcgcttctattctccgaaaacaaatactccacacaaagaagcgtatga
attaattaacaaattatggcaatacccatctccaaatttggtgatttcagtacttggaggtctgaaaaat
caacttttcaaaaatagtcaacttgaaagaaacttcaaaagaaatttatgcgagataatgaaaacaagta
caacttggattattggatctgggctagatgtcggtgtaagtaagatagttggcgaaactatcggtgcata
taggcaaggatctcgaggtcaaaaccaagcttgccttatcggggtgagttcttgcggagttgtgaaaaat
attgaaaagttaatttcaaacaattgcacgtatcctagagcttctgagttctctaatgggacatttaatt
taaacgagttttatgattgttatttgttctgcgatgacggatctacaaacttatgccaaaatgataagga
atttctggtagactttgaaaaatatttgcaaaatatattaggagttccagtaatacggataatgcttggt
ggtgaaaggtgcgagctggGTTCCCTTTAGTGAGGGTTAATTCGCCCT

>Smed-TRPM-k
GCGATTTAGGTGACACTATAGAAGAGAACgcttatttgtcaggatcttggaatatttatgatatttgtgg
cattttagtttatatcatcactttttttctgagatgttttcttagtcaagatttatttattgtcactcgt
tgtttttattcagttacattgagtatgaattgccttcgaattatgcaatattattttatcagcccgaccc
ttggacctaaagtaataatgataggacgaatgatcgttgatatttttttctttttaagtatattttcgct

attcattatatcttatggtaccttagccattgccttgcaagataataggagtagagcaatgaatttatcg
ctgattactgaaattttctaccgtccatattttcagacttatggagaattgatgttggatgaatttcata
ctaatgatgaaaatataacagatattgaccgaacaattgggcgcatgacaattatttgtggttttgcagt
ctttctattacttacaaatatcctactaattaatttattaattgcaatgtttggacatactttcaacaaa
attcaagtggaatcccataagtttttctctttcatccaattacaacttgttgaagaatttcttgaaaagc
cattgctgccaccccctttaacttactacattatttgataacaggcattgtttattgcggaaacaggtg
ctgccacaagcaaacacgGTTCCCTTTAGTGAGGGTTAATTCGCCCT

>Smed-TRPM-l
GCGATTTAGGTGACACTATAGAAGAGAACgggtcgttgcttaaaccgaaagttataatttccattaacgg
aggaatagaagatattgattttgataaagaagtggaaaagcatctgaaacaaacctttaaggaatttctg
attgcagataaccaatgggttattggaactggattgaattgcggaattcacaaattgatcagcgaaactg
tgcaagaatcaagatttgataacgaggacaagcaaattaaccttattggattttataatttttctgagat
agaaagcaatcaaagtgttgtcaagacggataaaaaaagagaaattaaccatttatccaatttgaagtta
gatcctcattataattgcctgattttttcatgagtttctttatgataaacctaaaaaggagcttgagttac
gtaagaatttcgaaaaatattgttcagaattaaaagcattcacctttcaaattctttggggtggagatga
attatctctggattgggcgattaattcactaatgacccaaatatatgttgtagtgatgaaggggtcagga
aaaagggctgatgtcatatgctcaatgctcaataaaattgattctgctgacaacatcgagatggcaatac
aagaatattgtggcaatcttgaaggagatctagatcgtcttagaaatcagttaggagaactttggaaata
tagagacGTTCCCTTTAGTGAGGGTTAATTCGCCCT

>Smed-TRPM-m
GCGATTTAGGTGACACTATAGAAGAGAACggtacccacccttttgacatgctgccaattcgactcgatca
catatgggttattggtttgtcttatctgtcataatcttctctccgtcacagatttcccggatcaagtgtt
gggaaacatcagaaatttgaaagagttttgtgaaaaatactcgattgctgtcatgaatcgagcggtgatt
cgagattttacacccgataaaaaaattatttctggatttttaaacaaaccaaacattttagctggacaac
gaattttggacattgcatttctgtcgaagtcgacgaagtttctcagtttgccttcttgccaaaaagctct
aactcgactttggtatagaggcaccagaagatgtccagcgttgaaattttatatgcattcttttattcat
tgcgtttttctaggattgttttcctattttttaataataaacttctattcgaatatatcggtgatagaaa
tagtttgttatctctacggaataggatatttcatggaagagactcgtcagatatatatctacaggcaaaa
agactttctgaagtcatattttaaagaccctacaaattacatagacatcagtgcaactggatttatggta
tttgggttcattctcagggtgagtttacaaattcccaagcatcctgatcaaagtgaggctggactcttta
ttgaaagtacgaacgtcGTTCCCTTTAGTGAGGGTTAATTCGCCCT

>Smed-TRPM-n
GCGATTTAGGTGACACTATAGAAGAGAACcctggcttcatatcgccgttagatacgatagggacgtgaag
attttgtccttccttgtcaatgaaatacaaattgacattcgctggacggattctaacaaatttacagctc
tccatttggcctgtcagaaatccaattacattcccggtgagttggagattttaattaaagaatctaacca
attagtggcccgctctttcgatgatgttaccccccttgaattggcatgtgcagacatgagaaagttaaag
gacttgaagagaaatagaatcgtaaacatcaacgaactacttcaacatatcgataaagtttcttttggtt
tgccagaaactgaggacttctatttcaatgacaagatttcaacgaaacgaatgtggttaaatcttttgca
gaaaaatgacaaacacattctgagttactacatgaaaaaggatccaaaattttatctgaaatattacatg
accgttgctgaaatgacggaaattgtagatcttagtttagaaaacggccatggggtgttgacatatatca
aaaataaatatttggaaaataatgtaagtataggagagatatttgtctctttgacaggactaaacttaga
aaagattcagtcaaatgatgaaaacgagacaacatatgtttactatcgttcggcattgttcatgacatta
atcacaggccgttttgaactgGTTCCCTTTAGTGAGGGTTAATTCGCCCT

>Smed-TRPM-o
GCGATTTAGGTGACACTATAGAAGAGAACggcgccaatccgaactatgccgatgaattcggaacgacagt
tcttcattttgcaatcaaaggtgaacgacaagatgttattgttgctctgattgagtgcggagcaaacttt

caagaatgggaaagcgatggaataaatcttgttcagtggtgcataaggcatggattggttgaggtgttag
atatactttatgcgaaatatgatgtagatttaactctatcggataatttgggtcgaagcagtcttcattt
ggcatgtatgttcggtcaagtaaaagttgccgaatatattttagcaactggattgttaagtgttgacgaa
agagatgataatggttggacgcctttgcattatgcttgctcggaagctacgattagttttcgggaaatga
ttcaggttttattcaaatacaaggctgatccgattctcgtaacgaccaataataaaacatcattggatct
tgcttatgaagccggacgagcgcaggatttaattgatgttttgacaatacataagatgttgcaactcctc
atttctgttgatagcattgtggttggaaatattctagtcagagatccaaatttagcaaaccagagcaaga
cgagtgaagttgacaatatggagcgtcaaattactataaagcataaagaattccattcagcgaataaact
tcaagaaagacatgttacGTTCCCTTTAGTGAGGGTTAATTCGCCCT

>Smed–TRPM–p
GCGATTTAGGTGACACTATAGAAGAGAACgccgttttattatatcaaaaaccaaaatccgacaaccatgt
ggttaagtattctgaaatacgatgataaataccgacttcagtttatgatgaaagattctgaaaatttctt
tgacagattttatctgaaagagaaagaattttctgatgttttatgcaattgtcacgacaatggaaccgga
ttgctgcaatattgtaggaagcaattgtcagttgtgaccgaaagaaaatgtgagaatttattttgtaatt
taagtggactgaaaataacgaacaactcaaatgacgaggataaagtttatttcaagtcggccttatttct
agctcttttaactggaagattcgaattggttccttctttgctaatgtacagtcaattaaacgtgatttta
tatggaatgctgacgtgtttaatttgcagaactctttcaaaaatcaaaaaattcccggatcatgtaattg
aagaaataatgaaagtaaaacaattttgtgaagattattcaatagctgtgttaaataagaccgaagtccg
agactcgacaatggagaaaaaagtggttttcgaatatttaaaccaatcagaacgatttttaaacacacg
attttggaaattgcatttttcagtaagagttcaaaatttctgggattaccgtcatgccaaaaatcattgg
cgcaactatggaatccaaagGTTCCCTTTAGTGAGGGTTAATTCGCCCT

>Smed–TRPM–q
GCGATTTAGGTGACACTATAGAAGAGAACgcacaaaaatgacaaacaccgattaataaattcaatgaaaa
aagatccgaatttcaaaagtaaatattacattcaacctgacttgttcattgacattgtaatggcatgcca
tgaaaacgggcaaggaatcgtgacatatgttaaaaagcaattttcattaataagcaaaccaaatctccc
gaagagatctttaaacagttatccggattgaatctgaaagaatcagcccatctaaacgaaaacgtatttt
acacatcatcagtgtttctttccttaattaccggaagatttgaattggttccaactcttttgtcatattg
tcaattcgaaatcattcctttcggtttgatcatttgtcggatctgtcacaatttgactcacgtgaaaata
tttcccgatcaagtgctggagaacattttgaaattgaaagatttcagtgaaaagtacgcaatcgctgttt
tgaatcgcactgtgatccgagatacgacaatgaataaaaagtcaatttccaatttttaaacaaatcaaa
atatctcgacaaaacaattttagatattgcccatttggggaaatgtaaacgatttctcagtttgccttcg
tgtcaaggagctttcgatgagatttggtggaagaacaaccgaaaatgtccagcagttaaatatatgatcc
attctattattcacctggGTTCCCTTTAGTGAGGGTTAATTCGCCCT

>Smed–TRPM–r
GCGATTTAGGTGACACTATAGAAGAGAACgtacaaaacatcctgtcaaactaattagtgatgaaagaata
atgaattgtttacagatgaactggaccacggacctagtcgttgtgaattgtttactagattccatcggta
atgtgaataaatcgaattctagaattcatattccatttgaaatcgaaacagaaaagacatgtatttatta
ccgagtcccagagacgcaagccatgggttggatttattcacatctgtcaactttatcaaagattcaaaaa
cctgccgctcgaataataataggaaagttcaattaatgaaattcaacacgaacaaaatctcgtttgaagg
tttggtagggaaaggtttaattgtcggagaaccgaaatttgttctttctattaatggaggttttgattct
gaaggtaaattcacagaatgccaaaggatgattcttaagcagatgatttcggaactaattgaaaagaatg
agacgtacatcattggatccggattcaaagtaggaatttacaaattatttggcgagattgcacggcatgt
aaaactattgaattttctaaaagatttctgtgtgatcggaatcagttcctggaaatatctgctaaattcg
gaaaatattatcccagggcaaacaaattatcatgaagatgacaaattttcttctggaaagtttccgttag
acagcatttgtgatctcGTTCCCTTTAGTGAGGGTTAATTCGCCCT

>Smed–TRPM–s

GCGATTTAGGTGACACTATAGAAGAGAACggaatggcttatgaaaataataaatactactgatgattacg
ttgatgaaagttttcttacacgattatatcacgattcagtaaccggcaatgtcagttgcgtgaaggagct
tcggaaaagatatgataatttgtctggcctaaagtcgcaaactaatagtttttgttgtaaacgaaagcag
caaagtgcaggaatacctgaaaatgaatcatttgtgggaactagtgaagcaaataataaaaaatacatat
acatagaagagttattcatttgggcactactatccggctatacagaagtagcgtttgtattgtggaaatt
ttgcagtgatttaattggaacggctttaaaaggagctattaatttaaaaaaagtcgaaaacgattacaaa
aataaaaacttggattgttcagcagaaaaaattttcaaagaatcaaaaaagtatgagatgttggcatata
aaatcattgaaaccgcttatgacaaaagtccaatcagagctattgagttaattaatcggcccctagccag
atggggtaataagagctgccttcaaatatcagttaatgaagacctcaaagaattttgtagcaagagtgct
tgtgagcattatgcacgtttggcttggtgggataaagaacttccaaataatcgagggaaaaaaaaagaaa
aagcaattgaaagctcgGTTCCCTTTAGTGAGGGTTAATTCGCCCT

>Smed−TRPM−t
GCGATTTAGGTGACACTATAGAAGAGAACagcagattatatcagaaatgatgacaaatcaggatttctga
ctaattttgaaacttttgaaatcgaggacttttattctctttcggttgaaagtttgaaaaccgcaataac
ctggtgcagaacgaaaaaaatttatttgattcatagaattatcaaaagaacccaaaacgttactaatctg
tcatttctttctgacaatcaaaacctccaattttacaagcaacatcatttattagattgtgacttcaaaa
cctggcttcatgttgcagtaatatctgagagccaccaaaatggagatgctaaaaatgtaactaattatgt
ggaaatagttgaatttttagtgagtaaactggaagttgacgtttctcgaacggattgtcacggatatacg
gctcttcattatgcttgtggaaacatgattttcaccaaagatttatacaatttgctaaatcatagttcga
aaaaaatcttgattttaacttcaaaagatgatctaactcccctagaaatcgcaagcagtgacattgagaa
attgaaaataataaaagacgtcgaaaaagaaggcttggatatcttattgtctgaaattgacagcatttcc
tcagcgataccgaagataaatgaaggtttcaagctctacgaaagtaaatcggcaactgaaatctggataa
tactgttaaacaataatgaccGTTCCCTTTAGTGAGGGTTAATTCGCCCT

Supplementary Table S1. TRPM homologs identified in *Schmidtea mediterranea*. Twenty-one different TRPM homologs (*TRPM-a1*, *-a2*, and *-b* to *-t*; left column) were identified through TBLASTN searches against *S. mediterranea* transcriptomes (first column on left). Corresponding Smes_v1 and dd_Smed_v6 identifiers are indicated (second and fifth columns, respectively). Top hit amongst human proteins (third column) identified through BLASTX searches using predicted full length cDNA contigs and corresponding E-values (fourth column). Distribution of expression of each TRPM homolog according to scRNA-seq analysis in asexual planarians (Plass et al., 2018; sixth column) and *in situ* hybridization analysis (ISH) in sexual planarians (this work; right column).

Putative TRPM	Match Smes_v1 contig	Reciprocal BLASTX vs human	E-value	Match dd_Smed_v6 contig (top match to Smes_v1)	Asexual Smed scRNAseq distribution (Plass/Planmine)	ISH result
**Smed-TRPM-a1 (DjTRPMa; E-val = 0)**	dd_Smes_v1_33400_1_1	TRPM3	5.0E-78	dd_Smed_v6_17857_0_1	GABA neurons	Peripheral neurons
**Smed-TRPM-a2 DjTRPMa; E-val = 2.04 × 10^{-135})**	dd_Smes_v1_28579_1_1	TRPM3	5.0E-71	dd_Smed_v6_26481_0_1	Goblet cells, GABA neurons	Intestine
**Smed-TRPM-b (DjTRPMb; E-val = 0)**	dd_Smes_v1_16865_1_1	TRPM3	8.0E-90	dd_Smed_v6_9288_0_1	Secretory 4, pharynx, epidermis, neoblasts	Brain
**Smed-TRPM-c**	dd_Smes_v1_41098_1_4	TRPM3	3E-172	dd_Smed_v6_11377_0_1	Glia, neoblasts	Testis
**Smed-TRPM-d**	dd_Smes_v1_16476_1_2	TRPM3	1.0E-102	dd_Smed_v6_13119_0_1	spp-11+ neurons, epidermis, pigment cells	-
**Smed-TRPM-e**	dd_Smes_v1_35607_1_1	TRPM3	6.0E-83	dd_Smed_v6_10029_0_1	Epidermis	-
**Smed-TRPM-f**	dd_Smes_v1_37543_1_1	TRPM5	6.0E-45	dd_Smed_v6_17981_0_1	ChAT neurons 1	Intestine, Brain, and peripheral neurons
**Smed-TRPM-g**	dd_Smes_v1_21604_1_3	TRPM1	1.0E-41	dd_Smed_v6_21927_0_1	Secretory 1, secretory 3, protonephridia	-
**Smed-TRPM-h**	dd_Smes_v1_41250_2_1	TRPM2	7.0E-84	dd_Smed_v6_11259_0_1	npp-18+ neurons, GABA neurons, cav-1+ neurons, secretory 4	Brain
**Smed-TRPM-i**	dd_Smes_v1_30648_1_2	TRPM2	8.0E-69	dd_Smed_v6_8493_0_1	otf+ cells 1, 2, glia, aqp+ parenchymal cells, ldlrr-1+ parenchymal cells	-
**Smed-TRPM-j**	dd_Smes_v1_33523_1_2	TRPM3	5.0E-61	dd_Smed_v6_18934_0_6	Epidermal progenitors, pgrn+ parenchymal cells, phagocytes	Head tip region
**Smed-TRPM-k**	dd_Smes_v1_32737_1_3	TRPM2	6.0E-74	dd_Smed_v6_7784_0_1	ldlrr-1+, pgrn+, and aqp+ parenchymal cells, phagocytes, glia	-
**Smed-TRPM-l**	dd_Smes_v1_89367_1_1	TRPM8	4.0E-31	dd_Smed_v6_6825_0_1	pgrn+ parenchymal cells, glia	Intestine and testis
**Smed-TRPM-m**	dd_Smes_v1_43220_1_1	TRPM1	6.0E-24	dd_Smed_v6_10717_0_1	Epidermal progenitors, epidermis, epidermis DVb, pharynx cell type	Brain
**Smed-TRPM-n**	dd_Smes_v1_4986_1_1	TRPM1	5.0E-16	dd_Smed_v6_13669_0_1	Epidermal progenitors, epidermis, epidermis DVb, pharynx cell type	Head tip region
**Smed-TRPM-o**	dd_Smes_v1_23507_1_1	TRPM3	6.0E-21	dd_Smed_v6_8382_0_1	otf+ cells 1, npp-18+ neurons	Intestine
**Smed-TRPM-p**	dd_Smes_v1_56329_1_1	TRPM3	1.0E-12	dd_Smed_v6_15270_0_1	Epidermal DVb neoblasts, phagocytes, psd+ cells, epidermis, secretory 1 cells	-
**Smed-TRPM-q**	dd_Smes_v1_10084_1_1	TRPM4	8.0E-11	dd_Smed_v6_26251_0_1	Epidermal DVb	-
**Smed-TRPM-r**	dd_Smes_v1_19870_1_4	TRPM5	1.0E-09	dd_Smed_v6_3620_0_1	Glia, aqp+ parenchymal cells	Brain and ventral nerve cords
**Smed-TRPM-s**	dd_Smes_v1_34165_1_3	TRPM6	2.0E-05	dd_Smed_v6_10485_0_1	Phagocytes	Intestine
**Smed-TRPM-t**	dd_Smes_v1_68197_1_1	TRPM3	2.0E-06	dd_Smed_v6_15098_0_1	Epidermis, goblet cells, protonephridia	-

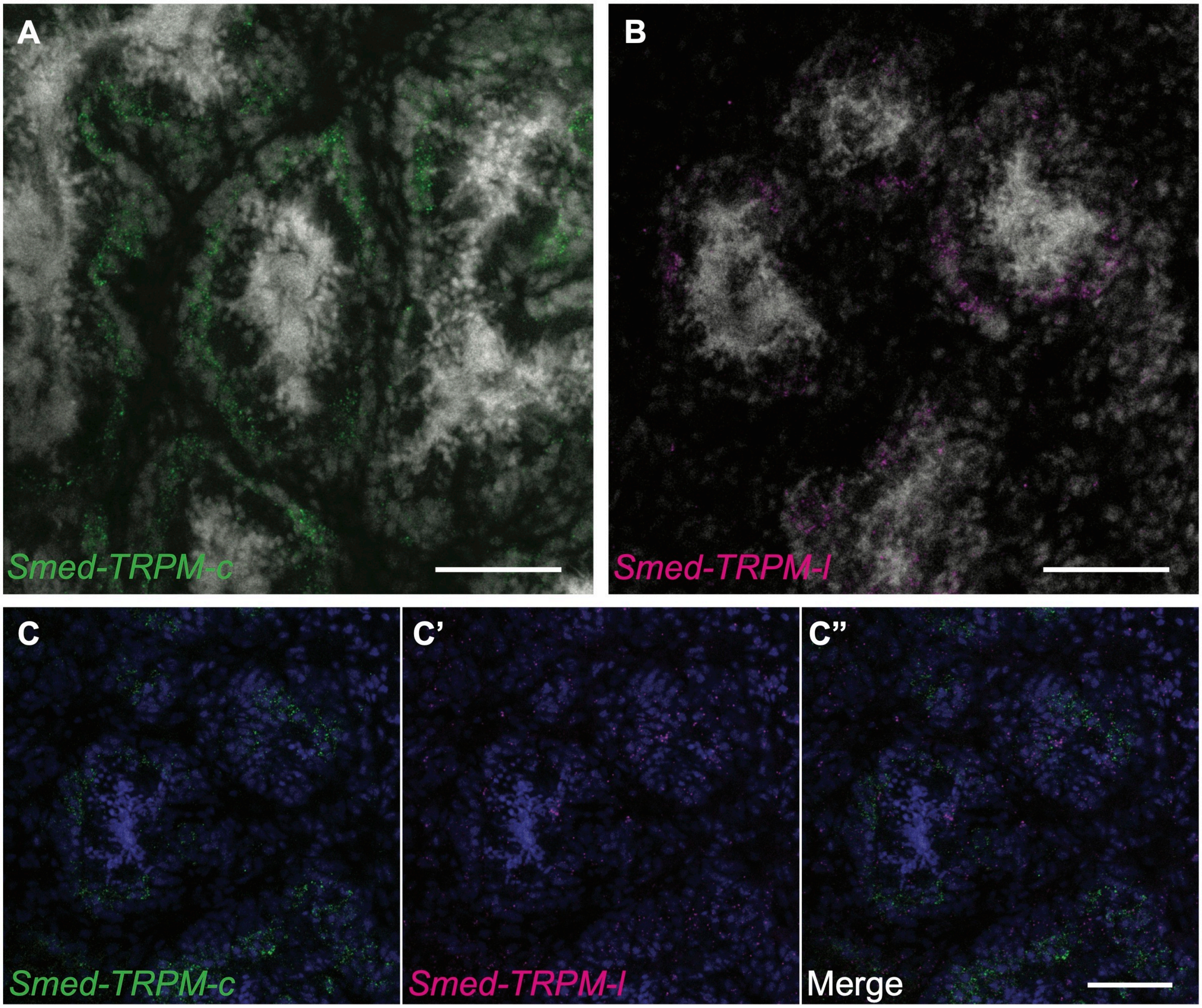

Supplementary Figure S1. Expression of *Smed-TRPM-c* and *Smed-TRPM-l* is detected in the planarian testes by fluorescence *in situ* hybridization (FISH). (A-B) Single confocal plane images taken with a 20X objective show detection of *Smed-TRPM-c* mRNA (A; green) and *Smed-TRPM-l* (B; magenta) mRNA in planarian testis lobes. DAPI staining (white) show distribution of cell nuclei. **(C)** Single confocal plane image shows partial overlap in expression of *Smed-TRPM-c* (green; C) and *Smed-TRPM-l* (magenta; C') in cells of planarian testis lobes detected by double-FISH. DAPI signal is shown in blue. Merged image shown in (C"). Scale bars = 50 μm.

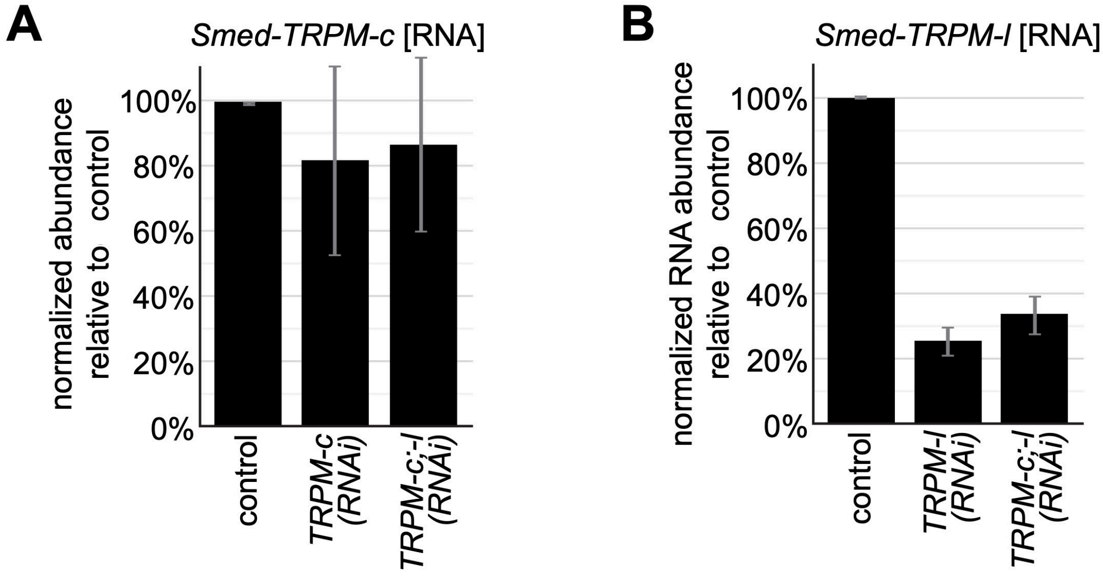

Supplementary Figure S2. *Smed-TRPM-c* and *Smed-TRPM-l* RNA levels decrease in planarians subjected to single and double RNAi. *Smed-TRPM-c* **(A)** and *Smed-TRPM-l* **(B)** RNA abundance measured by RT-qPCR in *Luciferase(RNAi)* (control; A and B), *Smed-TRPM-c(RNAi)* (*TRPM-c(RNAi)*; A), *Smed-TRPM-l(RNAi)* (*TRPM-l(RNAi)*; B), and *Smed-TRPM-c(RNAi);Smed-TRPM-l(RNAi)* double knockdowns (*Smed-TRPM-c;-l(RNAi)*; A and B). RNA abundance was assessed 1-week into RNAi treatments (on day 8, after feedings on day 1 and day 4). RNA levels for *Smed-TRPM-c* and *Smed-TRPM-l* were normalized to *beta-tubulin* RNA levels and shown as a fraction of the signals detected in *Luciferase(RNAi)*. Averages from analyses using at least two primer combinations and from ≥ 5 individuals separately are shown along with error bars showing standard deviation from the mean.

A ***Smed-TRPM-c* expression according to scRNAseq by (Plass et al., 2018).**

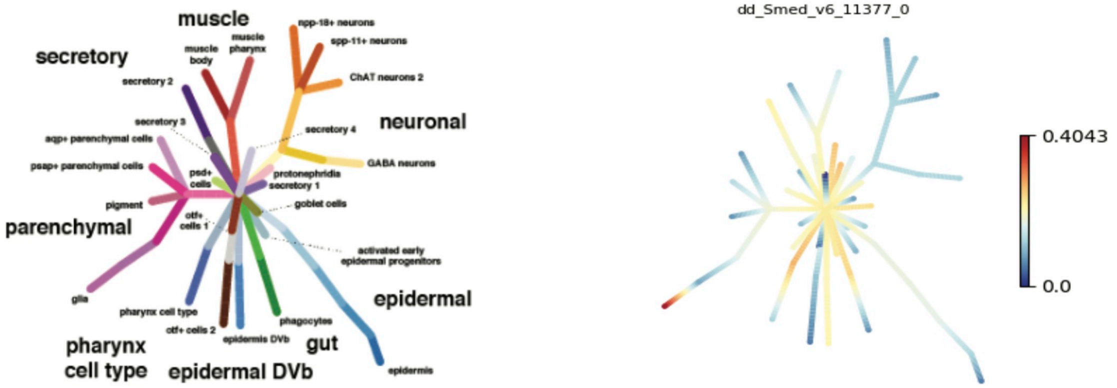

B ***Smed-TRPM-c* expression according to scRNAseq by (Fincher et al., 2018).**

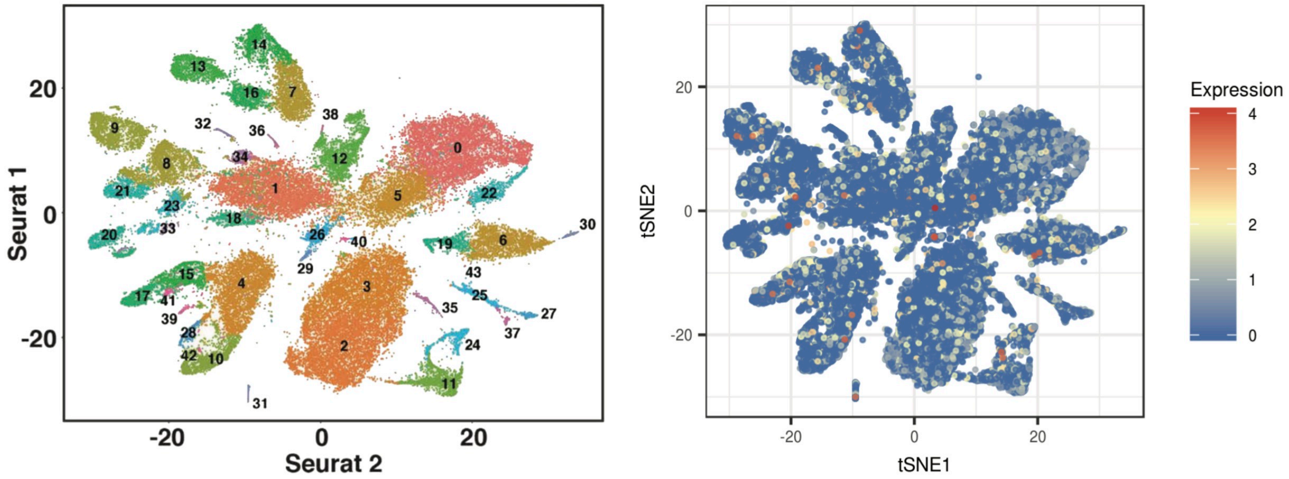

C ***Smed-TRPM-c* expression according to scRNAseq by (Zeng et al., 2018).**

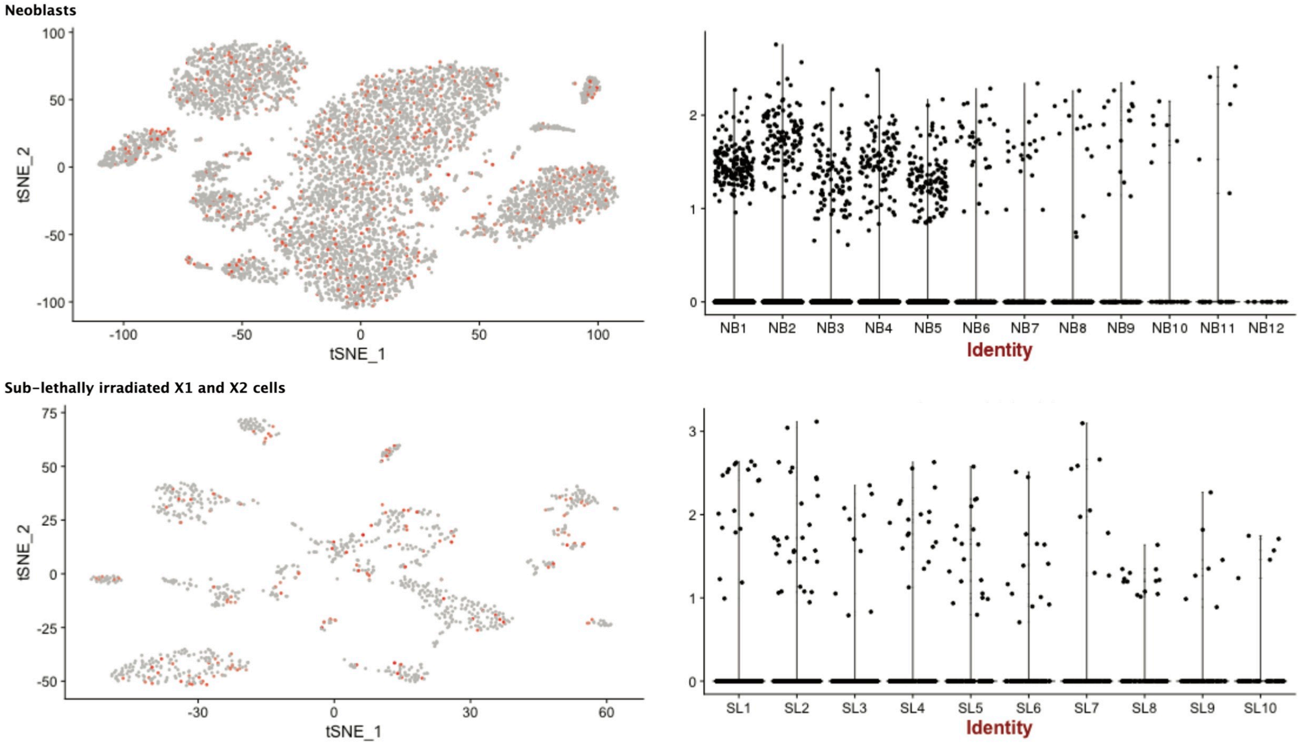

Supplementary Figure S3. Distribution of *Smed-TRPM-c* expression in asexual planarians according to single-cell RNAseq (scRNA-seq) studies. Images obtained from PlanMine (Rozanski et al., 2018) display enriched detection of *Smed-TRPM-c* in **(A)** glia (Plass et al.,2018) and **(B)** neoblasts (Fincher et al., 2018). **(C)** ScRNA-seq studies focused on planarian stem cells (Zeng et al., 2018) did not detect enrichment of *Smed-TRPM-c* detection in neoblasts.

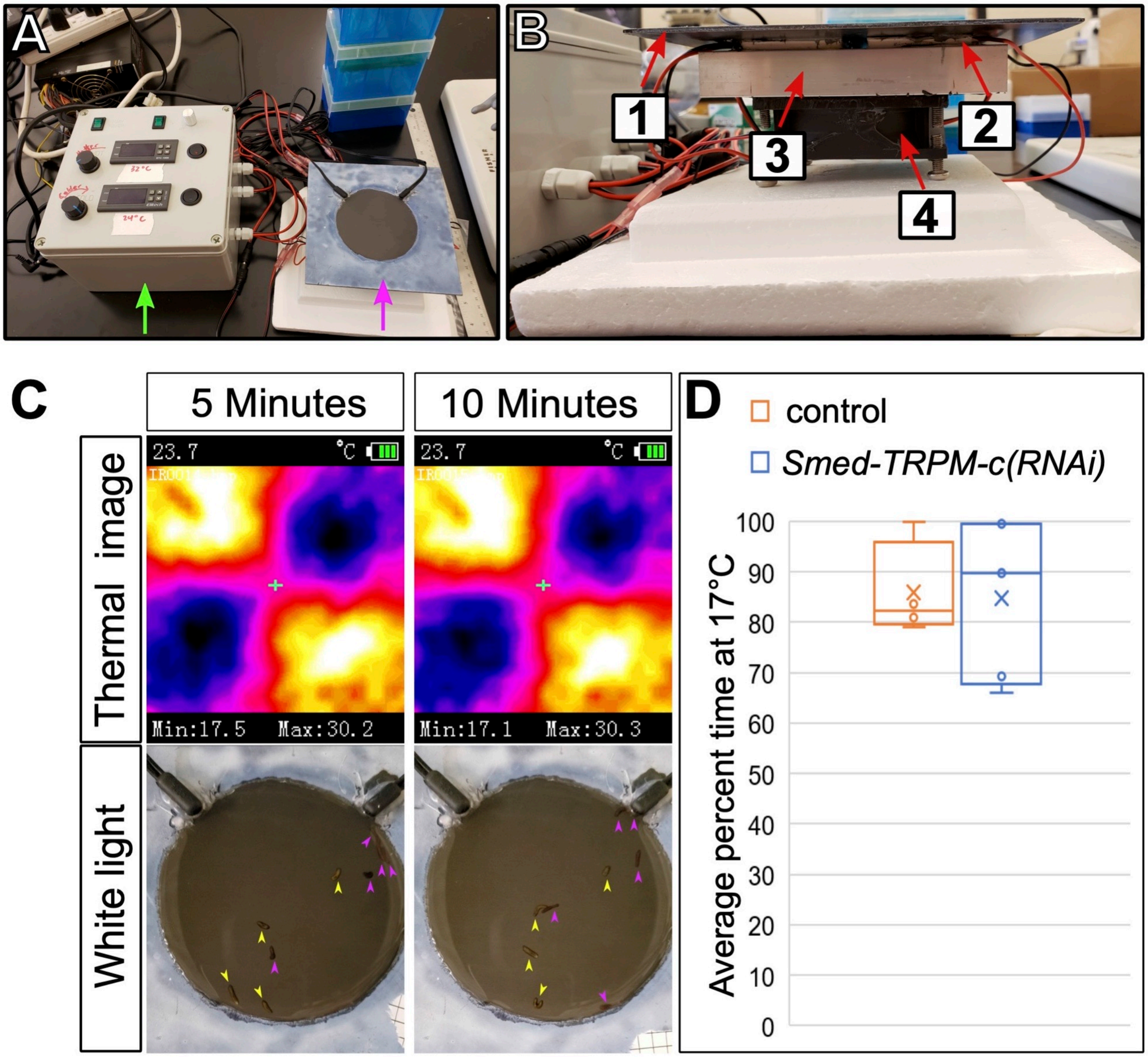

A
B
1
2
3
4
C
5 Minutes
10 Minutes
Thermal image
White light
23.7
Min:17.5 Max:30.2
Min:17.1 Max:30.3
D
control
Smed-TRPM-c(RNAi)
Average percent time at 17°C
100
90
80
70
60
50
40
30
20
10
0

Supplementary Figure S4. ***Smed-TRPM-c(RNAi)*** **does not exhibit deficits in planarian thermotactic behavior. (A)** Thermotaxis assay set-up inspired by Arenas et al. (2017) including the temperature display and control panel (green arrow) and the aluminum temperature plate with temperature probes attached (magenta arrow). **(B)** The temperature plate consists of an anodized aluminum plate (1); four Peltier plates (2), two with positive DC electric and two with negative DC electric; an aluminum heat sink for heat dispersion (3); and a fan to increase heat dispersion (4). **(C)** Heat map images of the temperature plate taken with a thermal camera at five and ten minutes into the assay recording (top). Snapshots of the thermotaxis plate under white light at the same timepoints (bottom) show asexual *luciferase(RNAi)* (yellow arrowheads) and *Smed-TRPM-c(RNAi)* (pink arrowheads) planarians in the cold (17°C) quadrants. **(D)** Box and whisker plot showing the range in the percentage of time spent in cold quadrants during the ten-minute experiment of control and *Smed-TRPM-c* knockdown planarians was not statistically significant. Mean is shown with an “x”. Median is shown with a horizontal line. Open circles represent the percentage for each individual planarian.

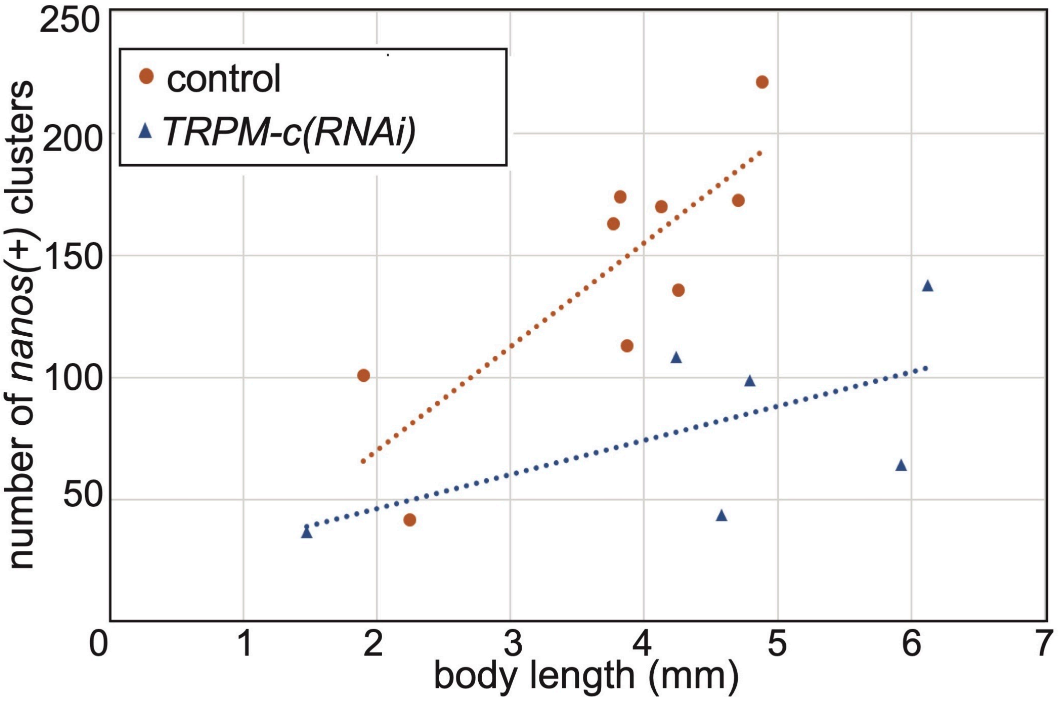

Supplementary Figure S5. *Smed-TRPM-c* RNAi results in decreased numbers of *nanos(+)* clusters in asexual planarians. Quantification of *nanos(+)* clusters (*y*-axis) observed by whole-mount *in situ* hybridization in asexual *Luciferase(RNAi)* and *Smed-TRPM-c(RNAi)* planarians plotted in relation to planarian body length (*x*-axis).

```
HTRPM2       59 LSSWIPENIKKKECVYFVESSKLSDAGK---------VVCQCGYTHEQH
HTRPM8       41 LVNFIQANFKKRECVFFTKDSKATEN------------VCKCGYAQSQH
HTRPM3       61 QKSWIERAFYKRECVHIIPSTKDPH-------------RCCCGRLIGQH
HTRPM6        3 QKSWIKGVFDKRECSTIIPSSKNPHRCTPVCQVCQNLIRCYCGRLIGDH
HTRPM7        3 QKSWIESTLTKRECVYIIPSSKDPHRCLPGCQICQQLVRCFCGRLVKQH
HTRPM1      -90 QKSWIEKTFCKRECIFVIPSMKDSN-------------RCCCGQFTNQH
HTRPM4        4 EQSWIPKIFKKKTCTTFIVDSTDPGG-----------TLCQCGRPRTAH
SmedTRPM-c   31 KYNWIDDNILKLECNRFHP--KSNG-------------YCACGRPAEEH
```

Supplementary Figure S6. Alignment of Human and Smed-TRPM-c TRPM Homology Regions (MHRs). MHRs of human TRPM channels (Grimm et al., 2003) were aligned using ClustalW 2.1 (Dereeper et al., 2008) and used as input for BoxShade (default settings). Identically conserved positions (black) and positions with changes that conserved similar amino acid properties (gray) in the majority of the sequences analyzed are highlighted.

```
HsTRPM2       751 NGLWRVTLCMLA-----FPLLLTGLISFREKRLQDV-----------------------
HsTRPM8       690 TKNWKIILCLFI-----IPLVGCGFVSFRKKPVDKH-----------------------
HsTRPM4       688 TPIWALVLAFFC-----PPLIYTRLITFRKSEEEPTREELEFDMDSVINGE---------
HsTRPM3       769 KNSGLKVILGIL-----LPPSILSLEFKNKDDMPYMSQAQEIHLQEKEAEEPEKPTKEKE
MmTRPM3       771 KNSGLKVILGIL-----LPPSILSLEFKNKDDMPYMTQAQEIHLQEKEPEEPEKPTKEKD
HsTRPM1       675 KNPGLKVIMGIL-----LPPTILFLEFRTYDDFSYQTS--------KENED----GKEKE
HsTRPM6       741 KNSWLKIIISII-----LPPTIITLEFKSKAEMSHVPQSQD-FQFMWYYSD--QNASSSK
HsTRPM7       755 KNSWYKVILSIL-----VPPAILLLEYKTKAEMSHIPQSQDAHQMTMDDSE--NNFQNIT
Smed-TRPM-c   718 KWVGLKVLIVLLSIIAIFPALPITILTLKSNRLIEFKTKQELALQPQTLEEYLNDSSSDS

HsTRPM2       782 ------------------------------------------------------------
HsTRPM8       721 ------------------------------------------------------------
HsTRPM4       734 GPVGTADPAEKTPLGVPRQSGR----------------------------------PGCCGG
HsTRPM3       824 EEDMELTAMLGRNNGESSRKKD----------------------------------EEEVQS
MmTRPM3       826 EEDMELTAMLGRSNGESSRKKD----------------------------------EEEVQS
HsTRPM1       718 EENTDANADAG------SRKGD----------------------------------EENEHK
HsTRPM6       793 ESASVKEYDLERGHDEKLDENQ----------------------------------HFGLES
HsTRPM7       808 EEIPMEVFKEVRILDSNEGKN-----------------------------------EMEIQM
Smed-TRPM-c   778 SSDSSSSDDETTDDAEAFGKSK-------------[...]--------------ENPQIS

HsTRPM2       782 ----GTPAARARAFFTAPVVVFHLNILSYFAFLCLFAYVLMVDFQPVP-SWCECAIYLWL
HsTRPM8       721 ----KKLLWYVVAFFTSPFVVFSWNVVFYIAFLLLFAYVLLMDFHSVP-HPPELVLYSLV
HsTRPM4       762 RCGGRRCLRRWFHFWGAPVTIFMGNVVSYLLFLLLFSRVLLVDFQPAPPGSLELLLYFWA
HsTRPM3       852 KHRLIPLGRKIYEFYNAPIVKFWFYTLAYIGYLMLFNYIVLVKMERWP-STQEWIVISYI
MmTRPM3       854 RHRLIPVGRKIYEFYNAPIVKFWFYTLAYIGYLMLFNYIVLVKMERWP-STQEWIVISYI
HsTRPM1       740 KQRSIPIGTKICEFYNAPIVKFWFYTISYLGYLLLFNYVILVRMDGWP-SLQEWIVISYI
HsTRPM6       821 GHQHLPWTRKVYEFYSAPIVKFWFYTMAYLAFLMLFTYTVLVEMQPQP-SVQEWLVSIYI
HsTRPM7       835 KSKKLPITRKFYAFYHAPIVKFWFNTLAYLGFLMLYTFVVLVQMEQLP-SVQEWIVIAYI
Smed-TRPM-c  1018 PGTQLSCRKKIYEFFYAPITKFYLNVISHILLIILLIRLAFIKWSVERIDYFELYIYVHI

HsTRPM2       837 FSLVCEEMRQLFYDPD-----------ECGLMKKAALYFSDFWNKLDVGAILLFVAGLTC
HsTRPM8       776 FVLFCDEVRQWYVN--------------------GVNYFTDLWNVMDTLGLFYFIAGIVF
HsTRPM4       822 FTLLCEELRQGLSGGGGSLASGGPGPGHASLSQRLRLYLADSWNQCDLVALTCFLLGVGC
HsTRPM3       911 FTLGIEKMREILMSEPG------------KLLQKVKVWLQEYWNVTDLIAILLFSVGMIL
MmTRPM3       913 FTLGIEKMREILMSEPG------------KLLQKVKVWLQEYWNVTDLIAILLFSVGMIL
HsTRPM1       799 VSLALEKIREILMSEPG------------KLSQKIKVWLQEYWNITDLVAISTFMIGAIL
HsTRPM6       880 FTNAIEVVREICISEPG------------KFTQKVKVWISEYWNLTETVAIGLFSAGFVL
HsTRPM7       894 FTYAIEKVREIFMSEAG------------KVNQKIKVWFSDYFNISDTIAIISFFIGFGL
Smed-TRPM-c  1078 ITNFLDHFRKFANTAGIN------------IAQKMKVHFESLWNFFDFSWGFYCLIAFSV

HsTRPM2       886 RLIP------------------------ATLYPGRVILSLDFILFCLRLMHIFTISKTLG
HsTRPM8       816 RLHSS---------------------NKSSLYSGRVIFCLDYIIFTLRLIHIFTVSRNLG
HsTRPM4       882 RLTP------------------------GLYHLGRTVLCIDFMVFTVRLLHIFTVNKQLG
HsTRPM3       959 RLQ-----------------------DQPFRSDGRVIYCVNIIYWYIRLLDIFGVNKYLG
MmTRPM3       961 RLQ-----------------------DQPFRSDGRVIYCVNIIYWYIRLLDIFGVNKYLG
HsTRPM1       847 RLQ-----------------------NQPYMGYGRVIYCVDIIFWYIRVLDIFGVNKYLG
HsTRPM6       928 RWG-----------------------DPPFHTAGRLIYCIDIIFWFSRLLDFFAVNQHAG
HsTRPM7       942 RFGAKWNFANA--------------YDNHVFVAGRLIYCLNIIFWYVRLLDFLAVNQQAG
Smed-TRPM-c  1126 RYFATYQFQQIKHQTDRNSTTLNSNNPSEIFLWGRNLIIILGSAYWVIKSLELMQNWRLFG

HsTRPM2       922 PKIIIVKRMMKDVFFFLFLLAVWVVSFGVAKQAILIH--NERRVDWLFRGAVYHSYLTIF
HsTRPM8       855 PKIIMLQRMLIDVFFFLFLFAVWMVAFGVARQGILRQ--NEQRWRWIFRSVIYEPYLAMF
HsTRPM4       918 PKIVIVSKMMKDVFFFLFFLGVWLVAYGVATEGLLRP--RDSDFPSILRRVFYRPYLQIF
HsTRPM3       996 PYVMMIGKMMIDMMYFVIIMLVVLMSFGVARQAILFP--NEEPSWKLAKNIFYMPYWMIY
MmTRPM3       998 PYVMMIGKMMIDMMYFVIIMLVVLMSFGVARQAILFP--NEEPSWKLAKNIFYMPYWMIY
HsTRPM1       884 PYVMMIGKMMIDMLYFVVIMLVVLMSFGVARQAILHP--EEKPSWKLARNIFYMPYWMIY
HsTRPM6       965 PYVTMIAKMTANMFYIVIIMAIVLLSFGVARKAILSP--KEPPSWSLARDIVFEPYWMIY
HsTRPM7       988 PYVMMIGKMVANMFYIVVIMALVLLSFGVPRKAILYP--HEAPSWTLAKDIVFHPYWMIF
Smed-TRPM-c  1186 AYLEMIRIMIKQIVPPVLILSVIMTAFGVVRQGIMYQGVVDLSIGNILKNIYLKPYFMLY

HsTRPM2       980 GQIPGYIDGVNFNPEHCSPNGTDPYKPKCPESDATQQRPAFPEWLTVLLLCLYLLFTNIL
HsTRPM8       913 GQVPSDVDGTTYDFAHCTFTGNE-SKPLCVELDEHN-LPRFPEWITIPLVCIYMLSTNIL
HsTRPM4       976 GQIP-QEDMDVALMEHSNCSSEPGFWAHPPGAQAGTCVSQYANWLVVLLLVIFLLVANIL
HsTRPM3      1054 GEVFADQID-------PPCGQNETREDGKI---IQLPPCKTGAWIVPAIMACYLLVANIL
MmTRPM3      1056 GEVFADQID-------PPCGQNETREDGKT---IQLPPCKTGAWIVPAIMACYLLVANIL
HsTRPM1       942 GEVFADQIDLYAMEINPPCGENLYDEEGK-----RLPPCIPGAWLTPALMACYLLVANIL
HsTRPM6      1023 GEVYAGEID--------VCSSQ--------------PSCPPGSFLTPFLQAVYLFVQYII
HsTRPM7      1046 GEVYAYEID--------VCANDSV-----------IPQICGPGTWLTPFLQAVYLFVQYII
Smed-TRPM-c  1246 GEVYAAEIDPVDFP-------------------EESRLTPLAWTVPLAMVLFMLSAVVV

HsTRPM2      1040 LLNLLIAMFNYTFQQVQEHTDQIWKFQRHDLIEEYHGRPAAPPPFILLSHLQLFIKRVVL
HsTRPM8       971 LVNLLVAMFGYTVGTVQENNDQVWKFQRYFLVQEYCSRLNIPFPFIVFAYFYMVVKKCFK
HsTRPM4      1035 LVNLLIAMFSYTFGKVQGNSDLYWKAQRYRLIREFHSRPALAPPFIVISHLRLLLRQLCR
HsTRPM3      1104 LVNLLIAVFNNTFFEVKSISNQVWKFQRYQLIMTFHERPVLPPPLIIFSHMTMIFQHLCC
MmTRPM3      1106 LVNLLIAVFNNTFFEVKSISNQVWKFQRYQLIMTFHERPVLPPPLIIFSHMTMIFQHVCC
HsTRPM1       997 LVNLLIAVFNNTFFEVKSISNQVWKFQRYQLIMTFHDRPVLPPPMIILSHIYIIIMRLSG
HsTRPM6      1061 MVNLLIAFFNNVYLDMESISNNLWKYNRYRYIMTYHEKPWLPPPLILLSHVGLLLRRLCC
HsTRPM7      1088 MVNLLIAFFNNVYLQVKAISNIVWKYQRYHFIMAYHEKPVLPPPLIILSHIVSLFCCICK
Smed-TRPM-c  1286 ILSLIIAGVTDIYGKMKESSVKVYMYLRYPIIIDYESRPFAPPPFIILVWIYILLKKMYF
```

Supplementary Figure S7. Alignment of mouse TRPM3, human TRPMs, and Smed-TRPM-c transmembrane regions. ClustalW 2.1 (Dereeper et al., 2008) alignment of mammalian TRPM homologs with Smed-TRPM-c transmembrane region reveals conserved sequence between transmembrane domains (red arrows) and serine residue (magenta arrow) known to be important in modulation of enzyme activity in response to PIP2 levels (Zhelay et al., 2018). Identically conserved positions (black) and positions with changes that conserved similar amino acid properties (gray) in the majority of the sequences analyzed are highlighted.

CLUSTAL multiple sequence alignment by MUSCLE (3.8)

```
NUDT9                              --------------------------------ENSHNKARTSPYPGSKVERSQVPNEKVG
HsTRPM2(NP_001307279.2)            SSEADVPTLASQKAAEEPDAEPGGRKKTEEPGDSYHVNARHLLYPNCPVTRFPVPNEKVP
DjTRPMa(AB845353.1)                --------------QKLGINKSNKQKFQIIKESDNHGEMFSQIIDDKNTREDKMRKTSMR
SmedTRPM-A1(dd_Smed_v6_17857)      --------------------------ILRKLGPNLPLNNEIKISKKNELMIEPAKKIEVT
SmedTRPM-F(dd_Smed_v6_17981)       -----------------VENLTTGRLIERIVNNHRLWRYQPFNFELYPGMRMKVPNNMVS
nvTRPM2(A7T1N0.1)                  -------------ALLQQQPPVKGQAAVPIQLTLLHYKARSSPYPGSTAKRFAVQDNMVD
                                                                                         .  :

NUDT9                              WLVEWQDYKPVEY-TAVSVLAGPRWADPQISESNFSPK--------FNEKD--GHVERKS
HsTRPM2(NP_001307279.2)            WETEFLIYDPPFY-TAERKDAAAM--DPMGDTLEPLSTIQ------YNVVD--GLRDRRS
DjTRPMa(AB845353.1)                LSDDMID-------DNSTKLWCCQIENHIILRLVPLVNLNQILKMKFINENEMESMTIQN
SmedTRPM-A1(dd_Smed_v6_17857)      SMLENTDYLPPGIVKKTLKLWKSKFVDHLICRFVPIFTAEQ--PVEFIEDDSKESQNEVL
SmedTRPM-F(dd_Smed_v6_17981)       WQIPYQDYNAFD--ASQEILLYPN--DQCFDADNPVIYKDS-----YNKYDNTNKLKRQS
nvTRPM2(A7T1N0.1)                  WQVPFPDYKPVNY-TAPVVLANPVWADKDLMAMSPRPELP------YNQMDHTCNVNRVS
                                                             :                   :   :

                                                                       # #
NUDT9                              KNGLYEIE--NGRPRNPAGRTGLVGRGLLGRWGPNHAADPIITRWK--RDSSGNKIXHPV
HsTRPM2(NP_001307279.2)            FHGPYTVQ--AGLPLNPMGRTGLRGRGSLSCFGPNHTLYPMVTRWR--RNEDGAICRKSI
DjTRPMa(AB845353.1)                LKDEIKIF--DVAPVNPFENLNQFGKPLLPFWGINHCIHIIISRFIG-EKESGNISN---
SmedTRPM-A1(dd_Smed_v6_17857)      TEDLIEFP--NGIPINPHETLNKIGKPLLPFWGVNSCIHLLISRWKPWEKDDENDIY---
SmedTRPM-F(dd_Smed_v6_17981)       MLGQYALDPITCAPFNPTGRTGLKGRGLLPHWGPNHSIIMLFTRWN--RTNSGSPIHRKN
nvTRPM2(A7T1N0.1)                  YNGTYVVK--DGLPLNPMGRTGMQGRGLLGRFGPNHAADPVVTRWK--RTSAGVMLQGG-
                                     .   .      * **    .  *.  *  :* *     :.:*:     .

                                                               ##
NUDT9                              SGKHILQFVAIKRKDCGEWAIP------GGXVDPGEKISATLKREFGEEALNSLQKTSAE
HsTRPM2(NP_001307279.2)            --KKMLEVLVVKLPLSEHWALP------GGSREPGEMLPRKLKRILRQEHWPS-------
DjTRPMa(AB845353.1)                --KPELQVVAIRRHDN--IELPF----HNSFCLNNSCSSNNMKSIPKQYLNLISNQKQEN
SmedTRPM-A1(dd_Smed_v6_17857)      --KSILQVLGIGTTNH--IELPFIIVQHNTECEQNLCSSSKLEQIIRDYFIEINKKKADN
SmedTRPM-F(dd_Smed_v6_17981)       --KRMMQYIALERANR--YGIPWFLVDHNSGCELNECSNKTAITFLDQRLQASYSGKKYE
nvTRPM2(A7T1N0.1)                  --KKVLEFVAIQRKDNNQWAIP------GGMVEPGQLVTQALKAEFGEEAMAKLNVSQEE
                                     *  :: : :         :*      .     .            :

                                                           # # #
NUDT9                              KREI-EEKLHKLFSQDHLVIYKGYVDDPRNTDNAWXETEAVNYH-DETGEIXDN---LXL
HsTRPM2(NP_001307279.2)            --------FENLLKCGM-EVYKGYMDDPRNTDNAWIETVAVSVHFQDQNDVELNRLNSNL
DjTRPMa(AB845353.1)                SSK--LNDFDLFNTVDMNEIYRGYICDFRNTNNSWMETTACNIHQTEIHKLSDDF--LML
SmedTRPM-A1(dd_Smed_v6_17857)      KTFLNEKEFESLNNSKYDKVYEGYLKDFRNSDNSWIETSAFNIHLEDNLEFTKPI--LHM
SmedTRPM-F(dd_Smed_v6_17981)       FLR------KKIVAATCTQIFKGFLDDHLNADNAWVETVVINFHESDKFHFTDDIL-KIF
nvTRPM2(A7T1N0.1)                  KERI-AKQIERLFQQGQ-EIYKGYVDDPRNTDNAWMETVAVNFH-DDKGDLFGD---ITL
                                              :       :: *:: *  *::*:* ** . . *  :   .        :

                                                          #
NUDT9                              EAGDDAGKVKWVDINDKLKLYASHSQFIKLVAEKRDAHWSEDSEADCHAL
HsTRPM2(NP_001307279.2)            HACDSGASIRWQVVDRRIPLYANHKTLLQKAAAEFGAHY-----------
DjTRPMa(AB845353.1)                FEQDEFEEGCWFNVNNELWTNPSNKDLLSKLSIYHDYVV-----------
SmedTRPM-A1(dd_Smed_v6_17857)      LQHDDSETLCWVDVDKSHWTNPSNIILLSFLSNFHGFEF-----------
SmedTRPM-F(dd_Smed_v6_17981)       VEINSPETVKWMDLAHSVNLRTSHDMILKTISQRMDAFF-----------
nvTRPM2(A7T1N0.1)                  QAGDDAAAVRWQRVSGNIPLYASHVSILEKVAKMRDAAF-----------
                                      :.     *  :       ..:  ::.  :   .
```

Supplementary Figure S8. Alignment of NUDIX homology regions in human TRPM2, *Nematostella vectensis* TRPM2, and planarian TRPMs. Muscle Multiple Sequence Alignment (MSA) software output from EMBL-EBI (Madeira et al., 2024) using default settings shows conserved residues in regions of homology to the hydrolase domain in human NUDT9 (NCBI: pdb|1Q33|A), human TRPM2 (NCBI: NP_001307279.2), *Nematostella vectensis* TRPM2 (NCBI: A7T1N0.1), *Dugesia japonica* TRPMa (NCBI: AB845353.1), as well as Smed-TRPM-A1 (dd_Smed_v6_17857) and Smed-TRPM-f (dd_Smed_v6_17981). Positions highlighted in yellow indicate active site residues identified in the Human ADP-ribose pyrophosphatase NUDT9 structure (Shen et al., 2003). Regions conserved between Human NUDT9, Human TRPM2 and *E. coli* ADP-ribose pyrophosphatase include a P-loop (gray) and the Nudix box motif (blue), the latter of which has largely diverged in planarians. Non-standard amino acids are represented by an “X”.
